# Consecutive cross-coupling reactions of 2,2-difluoro-1-iodoethenyl tosylate with boronic acids: efficient synthesis of 1,1-diaryl-2,2-difluoroethenes

**DOI:** 10.3762/bjoc.9.286

**Published:** 2013-11-14

**Authors:** Ju Hee Kim, Su Jeong Choi, In Howa Jeong

**Affiliations:** 1 Department of Chemistry & Medical Chemistry, Yonsei University, 1 Yonseidae-gil, Wonju, Gangwondo 220-710, Republic of Korea

**Keywords:** boronic acids, cross-coupling reaction, 1,1-diaryl-2,2-difluoroethene, 1,1-difluoro-1,3-dienes, 2,2-difluoro-1-iodoethenyl tosylate, organo-fluorine

## Abstract

The cross-coupling reactions of 2,2-difluoro-1-iodoethenyl tosylate (**2**) with 2 equiv of boronic acids in the presence of catalytic amounts of Pd(OAc)_2_ and Na_2_CO_3_ afforded the mono-coupled products **3** and **5** in high yields. The use of 4 equiv of boronic acids in the presence of catalytic amount of Pd(PPh_3_)_2_Cl_2_ and Na_2_CO_3_ in this reaction resulted in the formation of symmetrical di-coupled products **4** in high yields. Unsymmetrical di-coupled products **4** were obtained in high yields from the reactions of **3** with 2 equiv of boronic acids in the presence of catalytic amounts of Pd(OAc)_2_ and Na_2_CO_3._

## Introduction

The synthesis of 2,2-disubstituted-1,1-difluoroethenes have received much attention to synthetic organofluorine chemists in recent years because of their unique chemical reactivities toward nucleophiles to produce monofluorinated organic compounds [1–4], and their biological activity, such as mechanism-based enzyme inhibitors, in the area of medicinal chemistry [5–8]. The 1,1-difluoroethenylidene functionality in these compounds is also known to act as a bioisostere for the carbonyl group of many biologically active compounds [9–12]. Although numerous methods for the preparation of 2,2-disubstituted-1,1-difluoroethenes have been reported in the previous literature [13–22], a consecutive cross-coupling reaction of a proper precursor such as a 1,1-difluoroethenylidene species bearing a metal functional group, a halogen substituent or a tosylate group at the vinyl carbon will provide a concise and efficient method for the synthesis of 2,2-disubstituted-1,1-difluoroethenes. Burton et al. reported a straightforward method for the preparation of 1,1-diaryl-2,2-difluoroethenes from the consecutive cross-coupling reaction of the 2,2-difluoro-1-bromoethenylzinc reagent with aryl iodides, followed by arylboronic acids [17]. Recently, we also prepared 2,2-difluoro-1-tributylstannylethenyl tosylate and (2,2-difluoroethenylidene)bis(tributylstannane) which were utilized in the palladium-catalyzed consecutive cross-coupling reactions with electrophilic aryl iodides or nucleophilic arylstannane reagents to afford the corresponding 1,1-diaryl-2,2-difluoroethenes [20–21]. However, these previous reagents still have some drawbacks such as the existence of the toxic tributylstannyl group, thermal instability of ethenylzinc reagents and the use of at least one nucleophilic reactive site for the coupling partner. In contrast to these reagents, the 1,1-difluoroethenylidene species bearing both an electrophilic halogen substituent and a tosylate group at the vinyl carbon have not been studied in the cross-coupling reaction with stable and less toxic nucleophilic metal reagents such as aryl- and alkenylboronic acids. Herein, we report a preparation of 2,2-difluoro-1-iodoethenyl tosylate and its cross-coupling reactions with aryl- and alkenylboronic acids to give the corresponding 1,1-difluoroalkenes.

## Results and Discussion

**Scheme 1:**
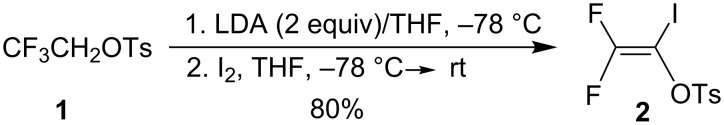
Preparation of 2,2-difluoro-1-iodoethenyl tosylate (**2**).

Although the chemistry of the 2,2-difluoroethenylidene species as a building block has been well established in recent years, 2,2-difluoro-1-iodoethenyl tosylate (**2**) was not previously prepared. However, we easily synthesized the starting material **2** from the reaction of 2,2,2-trifluoroethyl tosylate (**1**) with 2 equiv of LDA in THF at −78 °C, followed by treatment with 1 equiv of iodine (Scheme 1).

First, we attempted the consecutive palladium-catalyzed cross-coupling reaction of **2** with different arylboronic acids to afford unsymmetrical 1,1-diaryl-2,2-difluoroethenes. Since the use of a proper base in the Suzuki–Miyaura reaction is an important factor to increase the yield of coupled product, we screened bases to get the optimized reaction conditions. When **2** was reacted with 1 equiv of phenylboronic acid in the presence of 5 mol % of Pd(OAc)_2_ and Cs_2_CO_3_ (2 equiv) in methanol at room temperature for 15 h, mono- and di-coupled products **3a** and **4a** were obtained in 21% and 10% yields, respectively, along with a small amount of the self-coupled product (less than 5%) and reduced product. The use of 2 equiv of phenylboronic acid in the same reaction increased the yield of **3a** (38%) and **4a** (19%). However, the use of high molecular amounts of Pd catalyst did not improve the yield of **3**. The same reaction was performed with K_2_CO_3_ instead of Cs_2_CO_3_ as a base to give **3a** and **4a** in 56% and 16% yields. The use of K_3_PO_4_ in this reaction provided similar results. Finally, the optimized reaction condition was achieved by using Na_2_CO_3_ as a base, in which only mono-coupled product **3a** was obtained in 92% yield along with the self-coupled product derived from the excess boronic acid. When the reaction was performed in the presence of 5% Pd(PPh_3_)_2_Cl_2_ or Pd(CH_3_CN)_2_Cl_2_ instead of Pd(OAc)_2_, di-coupled product **4a** was always formed in 6–13% yield. Optimization of the cross-coupling reaction of **2** with phenylboronic acid is summarized in Table 1.

**Table 1:** Optimization of the cross-coupling reaction of **2** with phenylboronic acid.

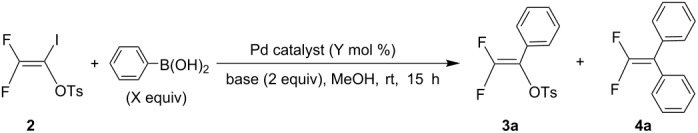						

Entry	Pd catalyst	X (equiv)	Y (mol %)	Base	Yield (%)^a^	

					3a	4a

1	Pd(OAc)_2_	1	5	Cs_2_CO_3_	21	10
2	Pd(OAc)_2_	2	5	Cs_2_CO_3_	38	19
3	Pd(OAc)_2_	3	5	Cs_2_CO_3_	18	40
4	Pd(OAc)_2_	2	10	Cs_2_CO_3_	30	24
5	Pd(OAc)_2_	2	5	K_2_CO_3_	56	16
6	Pd(OAc)_2_	2	5	K_3_PO_4_	54	17
7	Pd(OAc)_2_	2	5	Na_2_CO_3_	92	–^b^
8	Pd(PPh_3_)_2_Cl_2_	2	5	Na_2_CO_3_	78	6
9	Pd(CH_3_CN)_2_Cl_2_	2	5	Na_2_CO_3_	55	13

^a^Isolated yield. ^b^A trace amount of **4a** was obtained.

After the successful coupling reaction of **2** with phenylboronic acid under the optimized reaction conditions, the same reaction was performed with other arylboronic acids bearing a proton, fluoro, chloro, methyl, methoxy and trifluoromethyl on the *ortho*-, *meta*- or *para*-position of the benzene ring. Reactions were successful with both electron-donating and electron-withdrawing arylboronic acids to produce the corresponding 2,2-difluoro-1-arylethenyl tosylates **3** in excellent isolated yields. Especially, the coupling reactions with arylboronic acids having an electron-donating group at the *ortho*-position of the benzene ring also provided the corresponding coupled products **3l–n** in good yields. The cross-coupling reactions of **2** with arylboronic acids are summarized in Table 2.

**Table 2:** Preparation of 2,2-difluoro-1-arylethenyl tosylate **3**.

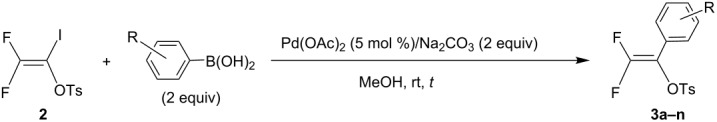			

Compound	R	*t* (h)	Yield (%)^a^

**3a**	H	15	92
**3b**	*p*-F	15	81
**3c**	*p*-Cl	14	84
**3d**	*p*-CH_3_	14	79
**3e**	*p*-OCH_3_	14	65
**3f**	*p*-CF_3_	15	85
**3g**	*m*-F	16	72
**3h**	*m*-Cl	16	76
**3i**	*m*-CH_3_	16	82
**3j**	*m*-OCH_3_	18	74
**3k**	*m*-CF_3_	18	80
**3l**	*o*-CH_3_	18	65
**3m**	*o*-OCH_3_	18	61
**3n**	*o*-Cl	18	68

^a^Isolated yield.

Direct diarylation reactions of **2** with arylboronic acids were also performed because di-coupled product **4a** was formed in a mono-arylation reaction (Table 1) and also a recent work showed a successful cross-coupling reaction of nonfluorinated enol tosylates with a variety of arylboronic acids [23]. We attempted a direct diarylation reaction of **2** with phenylboronic acid to establish the optimized reaction conditions. Initial reaction of **2** with 3 equiv of phenylboronic acid in the presence of 5 mol % of Pd(OAc)_2_ and 2 equiv of Na_2_CO_3_ in MeOH at room temperature for 22 h afforded the di-coupled product **4a** in 30% yield. The yield of **4a** was increased to 39% using 4 equiv of phenylboronic acid in this reaction. When the reaction was performed with K_2_CO_3_ or K_3_PO_4_ as a base under the same reaction conditions, the desired product **4a** was obtained in up to 49–52% yield. The use of more soluble Cs_2_CO_3_ resulted in the formation of **4a** in 61% yield. Increasing the reaction temperature did not improve the of yield of **4a**. We also examined the effect of the palladium catalyst in this reaction. Therefore, the same reaction was performed in the presence of 5 mol % of Pd(PPh_3_)_2_Cl_2_ instead of Pd(OAc)_2_, in which **4a** was obtained in 75% yield. Other palladium catalysts such as Pd(CH_3_CN)_2_Cl_2_ did not cause to improve the yield of **4a**. Optimization of the di-coupling reaction of **2** with phenylboronic acid is summarized in Table 3.

**Table 3:** Optimization of the di-coupling reaction of **2** with phenylboronic acid.

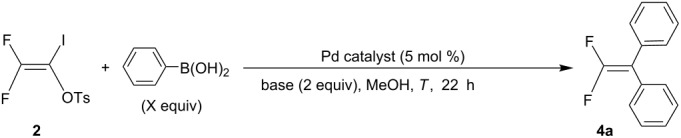					

Entry	Pd catalyst	X (equiv)	Base	*T* (°C)	Yield (%)^a^

1	Pd(OAc)_2_	3	Na_2_CO_3_	rt	30
2	Pd(OAc)_2_	4	Na_2_CO_3_	rt	39
3	Pd(OAc)_2_	4	K_2_CO_3_	rt	52
4	Pd(OAc)_2_	4	K_3_PO_4_	rt	49
5	Pd(OAc)_2_	4	Cs_2_CO_3_	rt	61
6	Pd(OAc)_2_	4	Cs_2_CO_3_	50	58
7	Pd(PPh_3_)_2_Cl_2_	4	Cs_2_CO_3_	rt	75
8	Pd(CH_3_CN)_2_Cl_2_	4	Cs_2_CO_3_	rt	21

^a^Isolated yield of **4a**.

Diarylation reactions of **2** with arylboronic acids substituted by fluoro, chloro, methyl, methoxy, nitro and trifluoromethyl substituent at the *meta*- or *para*-position of the benzene ring proceeded well under the optimized reaction conditions to give the corresponding symmetrical di-coupled products **4b–k** in 51–86% yield. However, coupling reactions with arylboronic acids having a substituent such as chloro, methyl or a methoxy group at the *ortho*-position of the benzene ring to produce the di-coupled products **4l–n** were unsuccessful. This result probably indicates that the coupling process could be affected by steric hindrance in the second coupling reaction step. The di-coupling reactions of **2** with arylboronic acids are summarized in Table 4.

**Table 4:** Preparation of symmetrical 1,1-diaryl-2,2-difluoroethenes **4**.

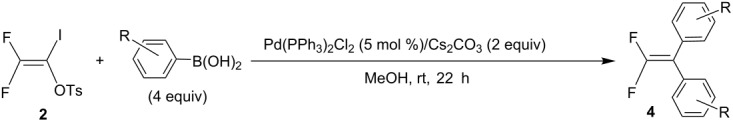		

Compound No	R	Yield (%)^a^

**4a**	H	75
**4b**	*p*-F	83
**4c**	*p*-Cl	86
**4d**	*p*-CH_3_	74
**4e**	*p*-OCH_3_	60
**4f**	*p*-NO_2_	73
**4g**	*m*-F	58
**4h**	*m*-Cl	51
**4i**	*m*-CH_3_	56
**4j**	*m*-OCH_3_	53
**4k**	*m*-CF_3_	60
**4l**	*o*-CH_3_	–^b^
**4m**	*o*-OCH_3_	–^b^
**4n**	*o*-Cl	–^b^

^a^Isolated yield. ^b^A trace amount of product was obtained.

Unsymmetrical 1,1-diaryl-2,2-difluoroethenes can be prepared from the coupling reaction of mono-coupled tosylates **3** with arylboronic acids under similar reaction conditions. Therefore, the cross-coupling reaction of **3a** with 2 equiv of *p*-chlorophenylboronic acid in the presence of 5 mol % Pd(PPh_3_)_2_Cl_2_ and 2 equiv of Cs_2_CO_3_ in MeOH at room temperature for 12 h afforded 1,1-diaryl-2,2-difluoroethene **4o** in 89% yield. The similar reactions of **3a** with arylboronic acids having a substituent such as fluoro, methyl, methoxy, and trifluoromethyl at the *meta*- or *para*-position of the benzene ring also provided the corresponding 1,1-diaryl-2,2-difluoroethenes **4p**–**v** in 75–90% yields. The coupling reaction between **3c** and *m*-methylphenylboronic acid also resulted in the formation of **4w** in 79% yield. Similarly, **3k** having an electron-withdrawing group on the benzene ring was also reacted with *p*-methylphenylboronic acid to yield **4x** in 70% yield. Table 5 summarizes the results of the coupling reactions of **3** with several arylboronic acids to give unsymmetrical 1,1-diaryl-2,2-difluoroethenes.

**Table 5:** Preparation of unsymmetrical 1,1-diaryl-2,2-difluoroethenes **4**.

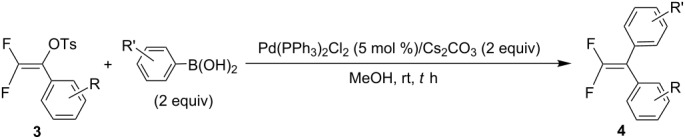				

Compound No	R	R’	*T* (h)	Yield (%)^a^

**4o**	H	*p*-Cl	12	89
**4p**	H	*p*-F	12	90
**4q**	H	*p*-CH_3_	12	81
**4r**	H	*p*-OCH_3_	14	79
**4s**	H	*p*-CF_3_	18	82
**4t**	H	*m*-Cl	12	81
**4u**	H	*m*-CH_3_	14	77
**4v**	H	*m*-CF_3_	14	75
**4w**	*p*-Cl	*m*-CH_3_	12	79
**4x**	*m*-CF_3_	*p*-CH_3_	12	70

^a^Isolated yield.

The successful cross-coupling reaction of **2** with arylboronic acids prompted us to examine similar coupling reactions with alkenylboronic acids. The same reaction conditions of the mono-coupling reaction of **2** with arylboronic acid was applied to the alkenylation of **2**. Therefore, the cross-coupling reaction of **2** with *trans*-2-phenylethenylboronic acid in the presence of 5 mol % of Pd(OAc)_2_ and 2 equiv of Na_2_CO_3_ in MeOH at room temperature for 15 h provided the cross-coupled product **5a** in 85% yield. The similar reaction of **2** with *trans*-oct-1-enylboronic acid afforded the cross-coupled product **5b** in 81% yield. The cross-coupling reactions of **2** with alkenylboronic acids are summarized in Table 6.

**Table 6:** The cross-coupling reactions of **2** with alkenylboronic acids.

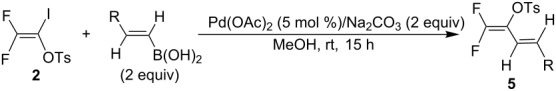		

Compound No.	R	Yield (%)^a^

**5a**	Ph	85
**5b**	CH_3_(CH_2_)_5_	81

^a^Isolated yield.

## Conclusion

In summary, we have successfully developed the consecutive cross-coupling reactions of 2,2-difluoro-1-iodoethenyl tosylate (**2**) with arylboronic or alkenylboronic acids in the presence of a suitable Pd catalyst and a base to afford 2,2-diaryl-1,1-difluoroethenes. The developed method provides synthetically useful advantages such as a straightforward procedure to give symmetrical and unsymmetrical 2,2-diaryl-1,1-difluoroethenes and the use of a less toxic reagent such as boronic acid.

## Supporting Information

File 1 Experimental details, full spectroscopic data and spectra.
