# Supplementary material for: Consecutive cross-coupling reactions of 2,2-difluoro-1-iodoethenyl tosylate with boronic acids: efficient synthesis of 1,1-diaryl-2,2-difluoroethenes
Source: Beilstein J Org Chem. 2013 Nov 14;9:2470–5. doi: 10.3762/bjoc.9.286 (PMC3869221; doi:10.3762/bjoc.9.286)
Supplement: File 1 — Experimental details, full spectroscopic data and spectra. [file Beilstein_J_Org_Chem-09-2470-s001.pdf]

`

Supporting Information

for

Consecutive cross-coupling reactions of 2,2-difluoro-1-iodoethenyl tosylate with boronic acids: efficient synthesis of 1,1-diaryl-2,2-difluoroethenes

Ju Hee Kim, Su Jeong Choi and In Howa Jeong*

Address: Department of Chemistry & Medical Chemistry, Yonsei University,

1 Yonseidae-gil, Wonju, Gangwondo 220-710, Republic of Korea

Email: In Howa Jeong* - jeongih@yonsei.ac.kr

* Corresponding author

Experimental details, full spectroscopic data and spectra.

`

Contents

**Instrumentation and chemicals**	S2
**General procedure for the synthesis of 2,2-difluoro-1-iodoethenyl *p*-toluenesulfonate (2)**	S3
**Spectral data for 2,2-difluoro-1-iodoethenyl *p*-toluenesulfonate (2)**	S3
**General procedure for the synthesis of 2,2-difluoro-1-arylethenyl *p*-toluenesulfonate 3 from 2,2-difluoro-1-iodoethenyl *p*-toluenesulfonate 2**	S3
**Spectral data for compounds 3**	S4
**General procedure for the synthesis of 1,1-diaryl-2,2-difluoroethenes 4 from 2,2-difluoro-1-iodoethenyl *p*-toluenesulfonate (2)**	S4
**Spectral data for compounds 4**	S5
**General procedure for the Synthesis of 2,2-difluoro-1-alkenylethenyl *p*-toluene sulfonate 5 from 2,2-difluoro-1-iodoethenyl *p*-toluenesulfonate (2)**	S6
**Spectral data for compounds 5**	S7
**^{1}H, ^{13}C and ^{19}F NMR spectra for new products**	S8
**References**	S34

Instrumentation and chemicals:

All solvents were dried by standard methods. Unless otherwise specified, chemicals were purchased from commercial suppliers and used without further purification. Column chromatography was performed on silica gel (230–400 mesh, Merck). TLC was performed on sheets pre-coated with silica gel (Kieselgel 60 PF254, Merck). Melting points were determined with a Fisher-Johns melting point apparatus and are uncorrected. The ^{1}H, ^{13}C and ^{19}F NMR spectra were performed on a Bruker 400 MHz NMR spectrometer, which operated at 400 MHz for ^{1}H nuclei, 100 MHz for ^{13}C nuclei and 376.5 MHz for ^{19}F nuclei and are internally referenced to residual protio solvent signals. Chemical shifts are reported in parts per million (ppm). Gas chromatography analyses were carried out on a Shimadzu gas chromatograph GC-14A instrument equipped with a flame ionization detector (FID) using nitrogen as a carrier gas. The column used for chromatography was a capillary column type (HP-530 m_250 mm) from Hewlett–Packard. Gas chromatography–mass spectrometry analyses were carried out on an Agilent Technologies G1530N

`

instrument (6890N Network GC system-5973 mass selective detector, EI, 70 eV). Microanalyses were performed on a CE instrument EA1110 elemental analyzer.

General procedure for the synthesis of 2,2-difluoro-1-iodoethenyl *p*-toluenesulfonate (2) : A 250 mL three-necked round-bottomed flask equipped with a magnetic stirring bar, septum and adaptor connected to an argon source was charged with 2,2,2-trifluoroethyl *p*-toluenesulfonate (3 g, 11.8 mmol) and 11 mL of dry THF. LDA (2.0 M solution, 13.0 mL, 26.0 mmol) was added dropwise to a stirred solution at −78 °C. After the addition was completed, the solution was stirred −78 °C for 30 min, and then iodine (3 g, 11.8 mmol) was added slowly to the stirred solution. The mixture was allowed to warm to room temperature over 2 h, then quenched with aqueous ammonium chloride(50 mL), the solution was extracted with ether (100 mL × 2), washed with 5% KF and brine, dried over anhydrous $MgSO_4$, and chromatographed on SiO_2 column. Elution with a mixture of hexane/ethylacetate (4:1) gave the desired product **2**.

Spectral data for 2,2-difluoro-1-iodoethenyl *p*-toluenesulfonate (2):

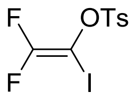

Light brown oil; 80% yield; ^{1}H NMR (400 MHz, $CDCl_3$): δ 7.84 (d, *J* = 8.4 Hz, 2H), 7.41 (d, *J* = 8.4 Hz, 2H), 2.48 (s, 3H); ^{19}F NMR (376 MHz, $CDCl_3$, internal standard $C_6H_5CF_3$) : δ -79.45 (d, *J* = 30.1 Hz, 1F), -94.82 (d, *J* = 30.1 Hz, 1F); ^{13}C NMR (100 MHz, $CDCl_3$): δ 155.3 (dd, *J* = 303, 283 Hz), 147, 131.7, 130.2, 129.1, 57.9 (dd, *J* = 56, 25 Hz), 21.9; MS m/z (relative intensity) 359 (M^+ , 2), 245 (2), 177 (25), 155 (100), 127 (42), 91 (79), 78 (25), 65 (31), 50(19); Anal. Calcd for $C_9H_7F_2O_3S$: C, 30.02; H, 10.55. Found: C, 29.91; H, 10.51.

General procedure for the Synthesis of 2,2-difluoro-1-arylethenyl *p*-toluenesulfonate 3 from 2,2-difluoro-1-iodoethenyl *p*-toluenesulfonate (2): A 25 mL two-necked round-bottomed flask equipped with a magnetic stirring bar, septum, and adaptor connected to an argon source was charged with **2** (0.3 g, 0.83 mmol), aryl boronic acid (1.67 mmol), $Pd(OAc)_2$ (5 mol %), Na_2CO_3 (1.67 mmol), and 12 mL of MeOH. After the reaction mixture was stirred at room temperature for 14–18 h, and then quenched with water. The reaction mixture was extracted with ether (15 mL × 2), washed with 5% KF and brine, dried over anhydrous $MgSO_4$, and chromatographed on a SiO_2 column. Elution with a mixture of ethylacetate/hexane (4:1) gave the desired product **3**.

`

Spectral data for comppounds 3:

The spectral data of compounds 3a–e and 3g–m are in accordance with reported data [1].

Spectral data 2,2-difluoro-1-(4-(trifluoromethyl)phenyl)ethenyl 4-methylbenzenesulfonate (3f):

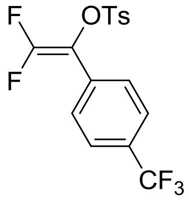

White solid; mp 58-59 ^{o}C; 85% yield; ^{1}H NMR (400 MHz, $CDCl_3$): δ 7.71 (d, *J* = 8.4 Hz, 2H), 7.50 (d, *J* = 8.4 Hz, 2H), 7.43 (d, *J* = 8.4 Hz, 2H), 7.22 (d, *J* = 8.4 Hz, 2H), 2.37 (s, 3H); ^{19}F NMR (376 MHz, $CDCl_3$, internal standard $C_6H_5CF_3$) : δ -63.98 (s, 3F), -87.78 (d, *J* = 30.1 Hz, 1F), -99.65 (d, *J* = 33.8 Hz, 1F); ^{13}C NMR (100 MHz, $CDCl_3$): δ 156.2 (dd, *J* = 297, 292 Hz), 146.1, 132.1, 132.0, 130.1, 129.7, 128.3, 126.6, 126.5, 123.4, 112.5 (dd, *J* = 41, 19 Hz), 21.4, 13.41; Anal. Calcd for $C_{16}H_{11}F_5O_3S$: C, 50.80; H, 2.93.

Spectral data 1-(2-chlorophenyl)-2,2-difluoroethynyl 4-methylbenzenesulfonate (3n):

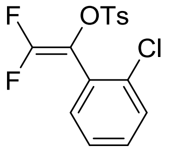

Yellow oil; 68% yield; ^{1}H NMR (400 MHz, $CDCl_3$): δ 7.58-7.55 (m, 3H), 7.33-7.22 (m, 5H), 1.26 (s, 3H); ^{19}F NMR (376 MHz, $CDCl_3$, internal standard $C_6H_5CF_3$) : δ -92.27 (d, *J* = 33.8 Hz, 1F), -99.67 (d, *J* = 33.8 Hz, 1F); ^{13}C NMR (100 Hz, $CDCl_3$): δ 160.3, 139.5, 137.4, 135.2, 132.4, 130.0, 128.7, 127.8, 125.3, 124.1, 115.4 (dd, *J* = 41, 19 Hz), 29.91; Anal. Calcd for $C_{16}H_{14}F_2O_4S$: C, 52.26 ; H, 3.22.

General Procedure for the synthesis of 1,1-diaryl-2,2-difluoroethenes 4 from 2,2-difluoro-1-iodoethenyl *p*-toluenesulfonate (2): A 10 mL two-necked round-bottomed flask equipped with a

`

magnetic stirring bar, septum and adaptor connected to an argon source was charged with $Pd(PPh_3)_2Cl_2$ (5 mol %), Cs_2CO_3 (0.56 mmol) and 5 mL of MeOH. After addition of arylboronic acid (1.11mmol) and **2** (0.28 mmol) (or **3** for the unsymmetrical products) the mixture was stirred at room temperature for up to 22 h and then quenched with water. The reaction mixture was extracted with ether (10 mL × 2), washed with 5% KF and brine, dried over anhydrous $MgSO_4$, and chromatographed on SiO_2 column. Elution with a petroleum ether or hexane gave the desired product.

Spectral data for compounds 4:

The spectral data of compounds 4a, o, q, r, u–w are in accordance with data reported in [1], the data of 4b–k were also already published in [2].

Spectral data 1-(2,2-difluoro-1-phenylvinyl)-4-fluorobenzene (4p):

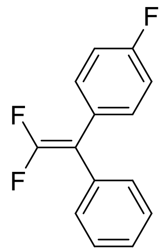

Colorless oil; 90% yield; ^{1}H NMR (400 MHz, $CDCl_3$): δ 7.37-7.01 (m, 9H); ^{19}F NMR (376 MHz, $CDCl_3$, internal standard $CFCl_3$): δ -88.81 (d, *J* = 34 Hz, 1F), -88.91 (d, *J* = 34 Hz, 1F);-115.16 (m, 1F); ^{13}C NMR (100 MHz, $CDCl_3$): δ 163.5, 161.1, 154.0 (t, *J* = 291 Hz), 134.3, 131.54, 130.5, 129.7, 128.7, 127.9, 115.8, 115.5 95.7 (t, *J* = 18 Hz), 129.8; Anal. Calcd for $C_{14}H_9F_3$: C, 71.79; H, 3.87. Found: C, 71.48; H, 3.81.

Spectral data 1-(2,2-difluoro-1-phenylvinyl)-4-(trifluoromethyl)benzene (4s):

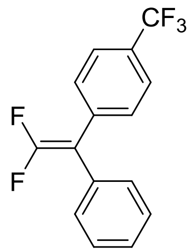

`

Colorless oil; 82% yield; ^{1}H NMR (400 MHz, $CDCl_3$): δ 7.60 (d, *J* = 8.0 Hz, 2H), 7.39-7.33 (m, 5H), 7.24 (d, *J* = 2.1 Hz, 2H); ^{19}F NMR (376 MHz, $CDCl_3$, internal standard $C_6H_5CF_3$) : δ -63.66 (s, 3F), -86.63 (d, *J* = 30.1 Hz, 1F), -87.36 (d, *J* = 30.1 Hz, 1F), -63.66 (s, 3F); ^{13}C NMR (100 MHz, $CDCl_3$): δ 154.3 (t, *J* = 293 Hz), 138.4, 133.6, 130.1, 129.9, 128.9, 128.2, 125.6 (dd, *J* = 76 Hz), 122.9, 95.9 (t, *J* = 17 Hz); Anal. Calcd for $C_{15}H_9F_5$: C, 63.39; H, 3.19. Found: C, 63.02; H, 3.22.

Spectral data 1-chloro-3-(2,2-difluoro-1-phenylvinyl)benzene (4t):

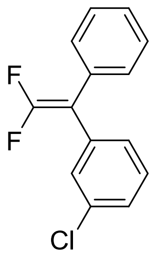

Yellow oil; 81% yield; ^{1}H NMR (400 MHz, $CDCl_3$): δ 7.71-7.69 (m, 2H), 7.25-7.19 (m, 6H), 2.40 (s, 3H); ^{19}F NMR (376 MHz, $CDCl_3$, internal standard $CFCl_3$) : δ -88.78 (d, *J* = 33.8 Hz, 1F), -99.85 (d, *J* = 33.8 Hz, 1F); ^{13}C NMR (100 MHz, $CDCl_3$): δ 156.3 (dd, *J* = 296, 291 Hz), 146.2, 134.6, 132.7, 130.2, 129.8, 128.9, 126.4, 124.6, 112.6 (dd, *J* = 41, 19 Hz), 21.8; Anal. Calcd for $C_{15}H_{12}F_2O$: C,67.08; H, 3.62. Found: C, 67.36; H, 3.53.

Spectral data 1-(2,2-difluoro-1-*p*-tolylvinyl)-3-(trifluoromethyl)benzene (4x):

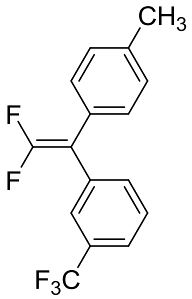

Colorless oil; 70% yield; ^{1}H NMR (400 MHz, $CDCl_3$): δ 7.67-7.44 (m, 6H), 7.19-7.12 (m, 2H), 2.37 (s, 3H); ^{19}F NMR (376 MHz, $CDCl_3$, internal standard $CFCl_3$): δ -63.72 (s, 3F), -87.61 (d, *J* = 30.8 Hz, 1F), -88.30 (d, *J* = 30.8 Hz, 1F); ^{13}C NMR (100 MHz, $CDCl_3$): δ 154.1 (t, *J* = 292 Hz), 140.8, 133.1, 130.8, 129.8, 129.6, 129.1, 126.5, 126.4, 124.9, 95.7 (t, *J* = 10 Hz), 29.9, 21.4; Anal. Calcd for $C_{15}H_{11}ClF_2$: C, 64.43; H, 3.72. Found: C, 64.66; H, 3.66.

`

General Procedure for the synthesis of 2,2-difluoro-1-alkenylethenyl *p*-toluenesulfonate (5) from 2,2-difluoro-1-iodoethenyl *p*-toluenesulfonate (2): A 10mL two-necked round bottomed flask equipped with a magnetic stirring bar, septum and an adaptor connected to an argon source was charged with $Pd(OAc)_2$ (5 mol %), Na_2CO_3 (0.56 mmol) and 5 mL of MeOH. After addition of alkenylboronic acid (5.56 mmol) and **2** (0.1g, 0.28 mmol), the mixture was stirred at room temperature for 15 h and then quenched with water. The mixture was extracted with ether (10 mL × 2), washed with 5% KF and brine, dried over anhydrous $MgSO_4$, and chromatographed on a SiO_2 column. Elution with a mixture of hexane/ethyl acetate (4/1) gave the desired product.

Spectral data for compounds 5:

Spectral data (E)-1,1-difluoro-4-phenylbuta-1,3-dien-2-yl 4-methylbenzenesulfonate (5a) :

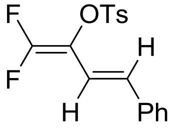

Colorless oil; 85% yield; ^{1}H NMR (400 MHz, $CDCl_3$): δ 7.94-7.92 (m, 2H), 7.38-7.26 (m, 9H), 6.58-6.48 (m, 2H), 2.44 (s, 3H); ^{19}F NMR (376 MHz, $CDCl_3$, internal standard $C_6H_5CF_3$) : δ -94.30 (d, *J* = 38 Hz, 1F), -105.20 (dd, *J* = 38 Hz, 1F); ^{13}C NMR (100 MHz, $CDCl_3$): δ 155.4 (dd, *J* = 298, 293 Hz, 1F), 146.2, 135.8, 133.1, 130.8, 130.7, 130.1, 128.8, 128.6, 128.5, 126.7, 114.0 (dd, *J* = 45, 18 Hz), 113.8, 21.7; MS m/z (relative intensity) 336 (M^+, 7), 181 (5), 155 (31), 131 (100), 91 (74), 65 (10); Anal. Calcd for $C_{14}H_{10}F_2$: C, 60.70; H, 4.1. Found: C, 60.36; H, 4.13.

Spectral data (E)-1,1-difluoro-4-phenylbuta-1,3-dien-2-yl 4-methylbenzenesulfonate (5b) :

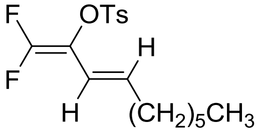

Yellow oil; 81% yield; ^{1}H NMR (400 MHz, $CDCl_3$): δ 7.85-7.83 (d, 2H), 7.36-7.34 (d, 2H), 5.85-5.69 (m, 2H), 2.46 (s, 3H), 2.03-2.00 (m, 2H), 1.30-1.25 (m, 8H), 0.89 (t, *J* = 6.8 Hz, 3H); ^{19}F NMR (376 MHz, $CDCl_3$, internal standard $C_6H_5CF_3$) : δ -91.3 (d, *J* = 30 Hz, 1F), 102.7 (dd, *J* = 30 Hz); ^{13}C NMR (100 MHz, $CDCl_3$): δ 154.8 (dd, *J* = 296, 290 Hz), 145.9, 134.2, 133.4, 130.0, 128.6, 115.6, 113.4 (dd, *J* = 43, 17 Hz), 113.2 (dd, *J* = 17 Hz), 32.9, 31.9, 29.0, 22.8, 21.9, 14.3; Anal. Calcd for $C_{14}H_{10}F_2$: C, 59.28; H, 6.44. Found: C, 58.91; H, 6.39.

`

^{1}H, ^{13}C and ^{19}F NMR spectra for products:

Spectra of compounds 3a–e, 3g–m and 4a, o, q, r, u–w were already reported [1].

Spectra of compounds 4b–k were also already published [2].

[^{1}H NMR spectrum of **2**]

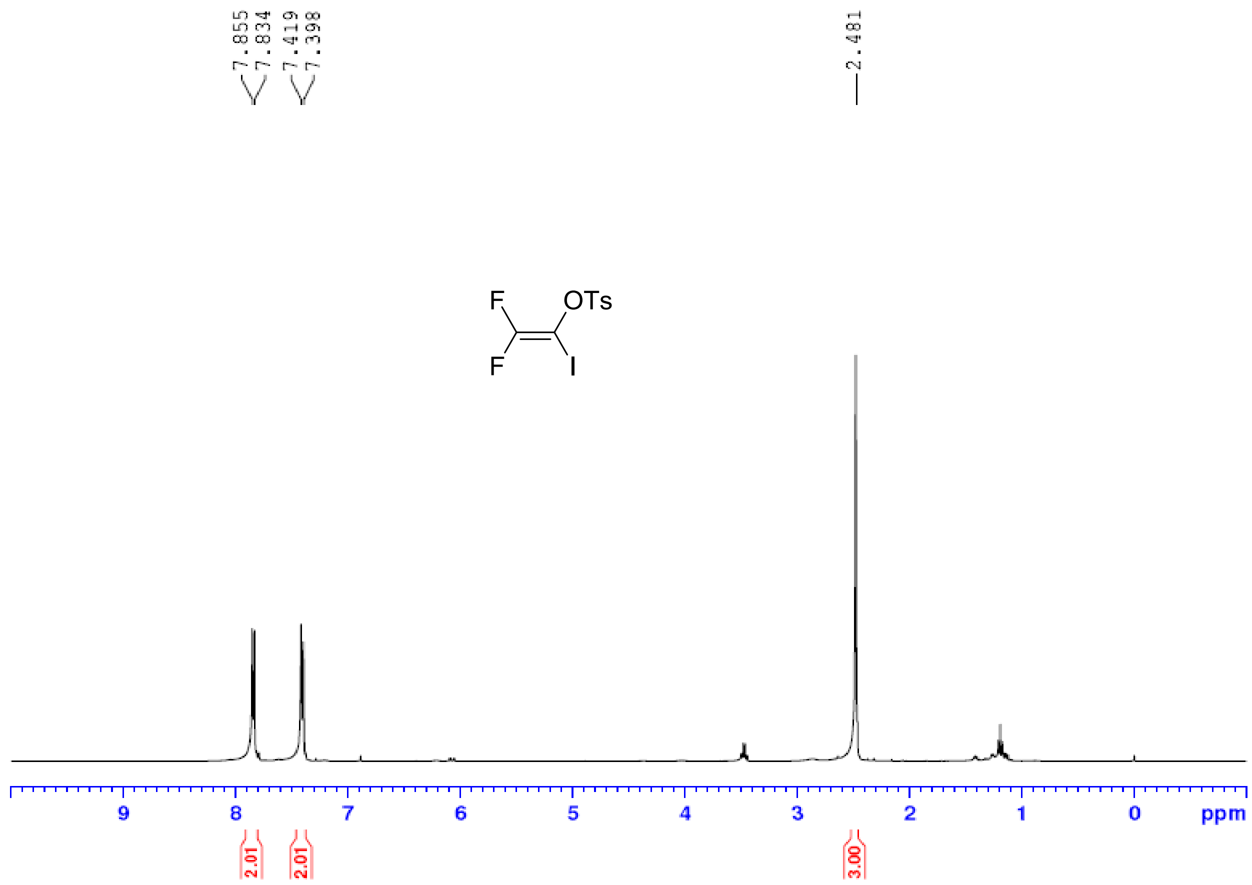

`

[^{13}C NMR spectrum of **2**]

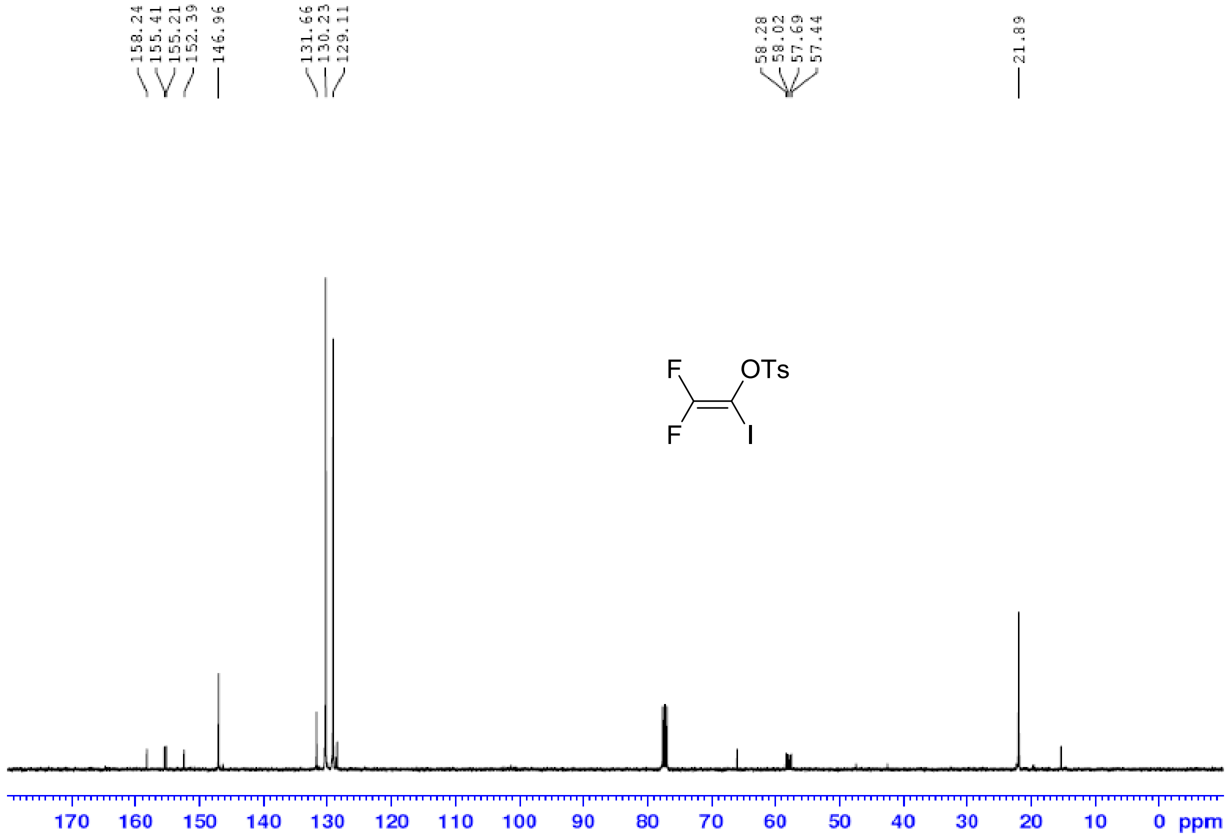

`

[^{19}F NMR spectrum of **2**]

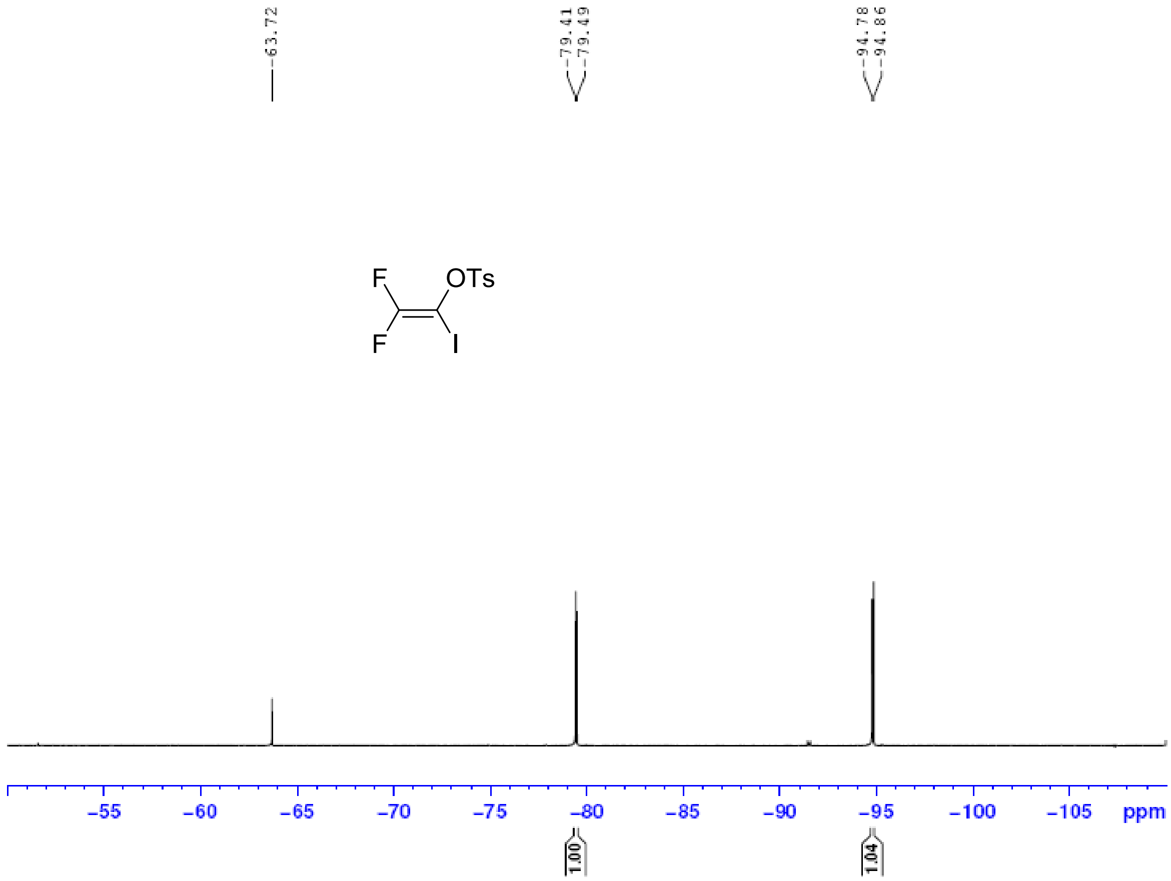

`

[^{1}H NMR spectrum of **3f**]

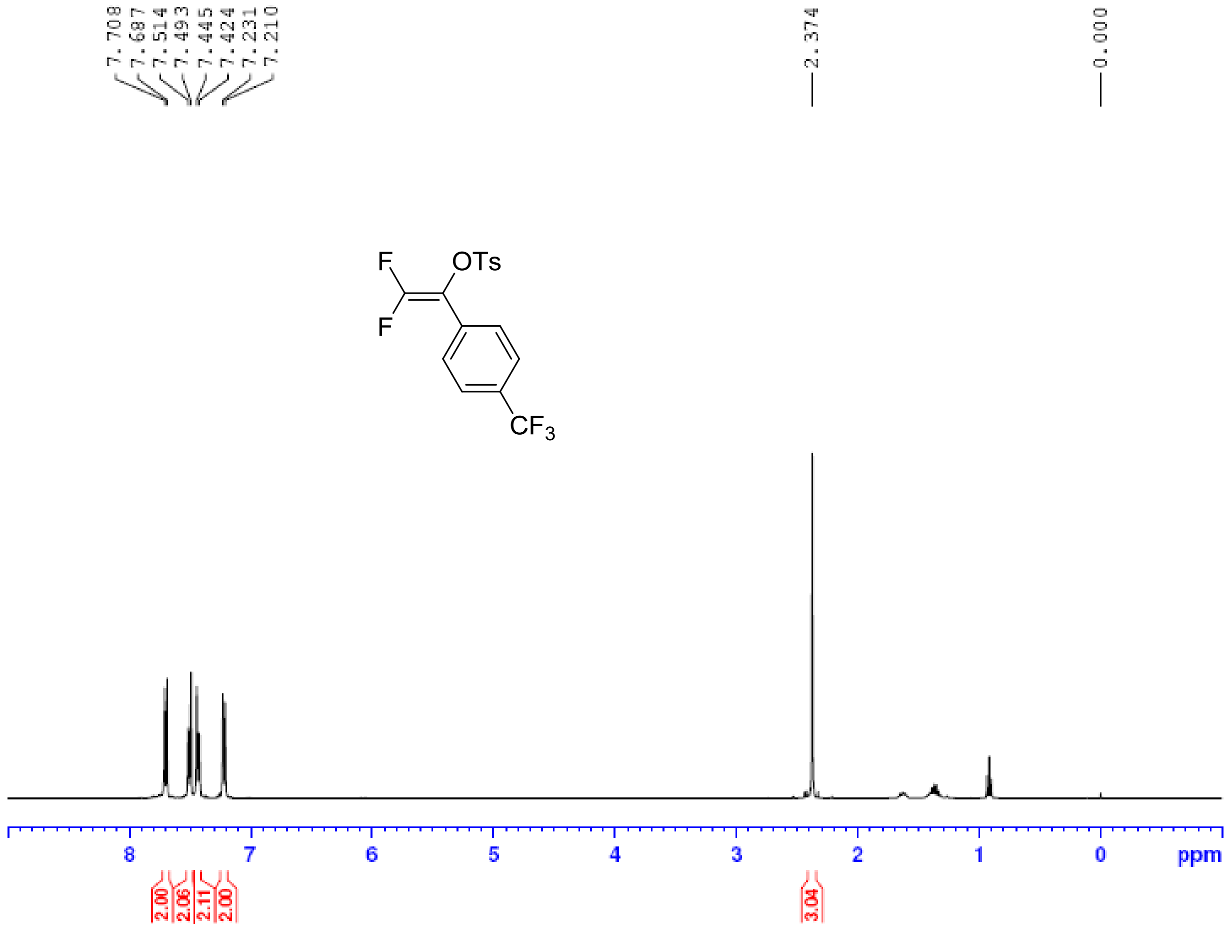

`

[^{13}C NMR spectrum of **3f**]

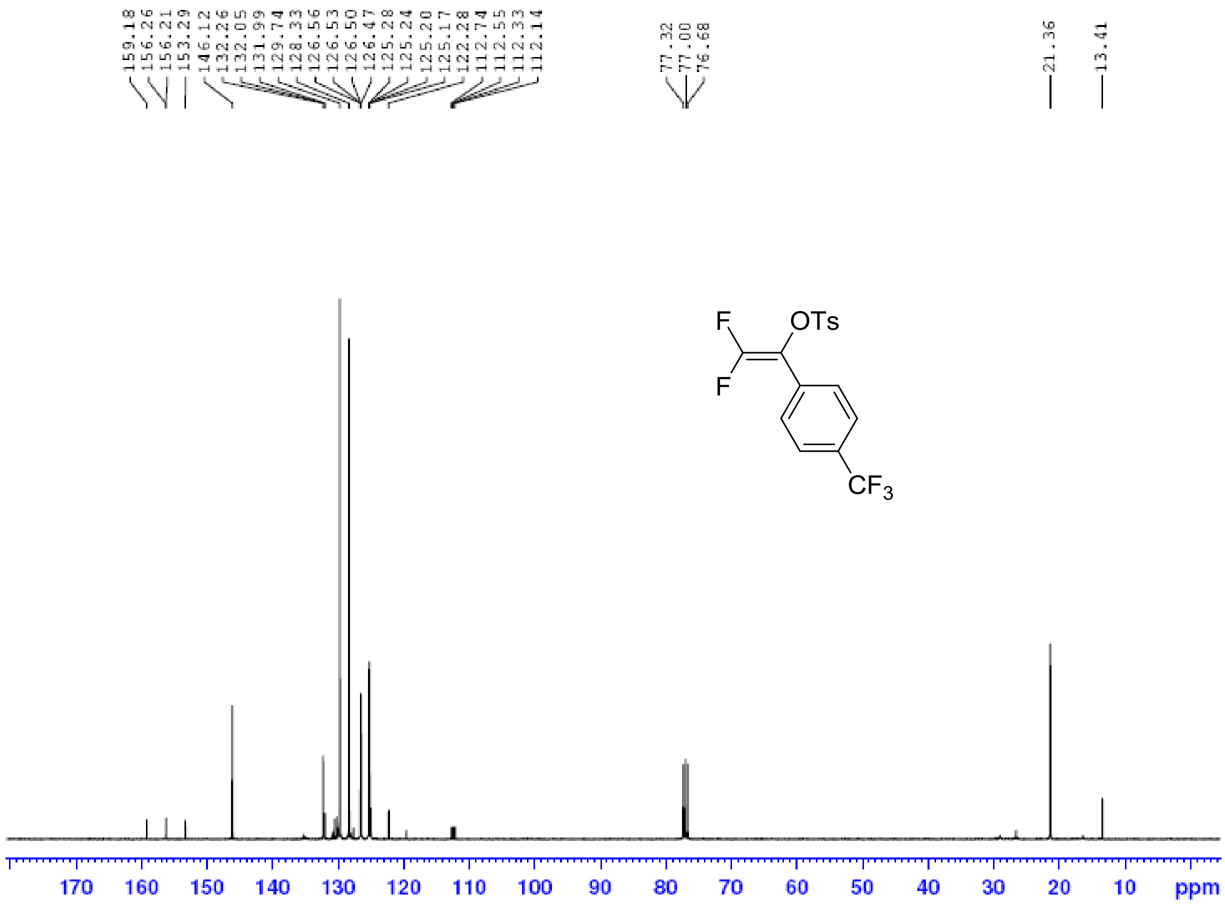

`

[^{19}F NMR spectrum of **3f**]

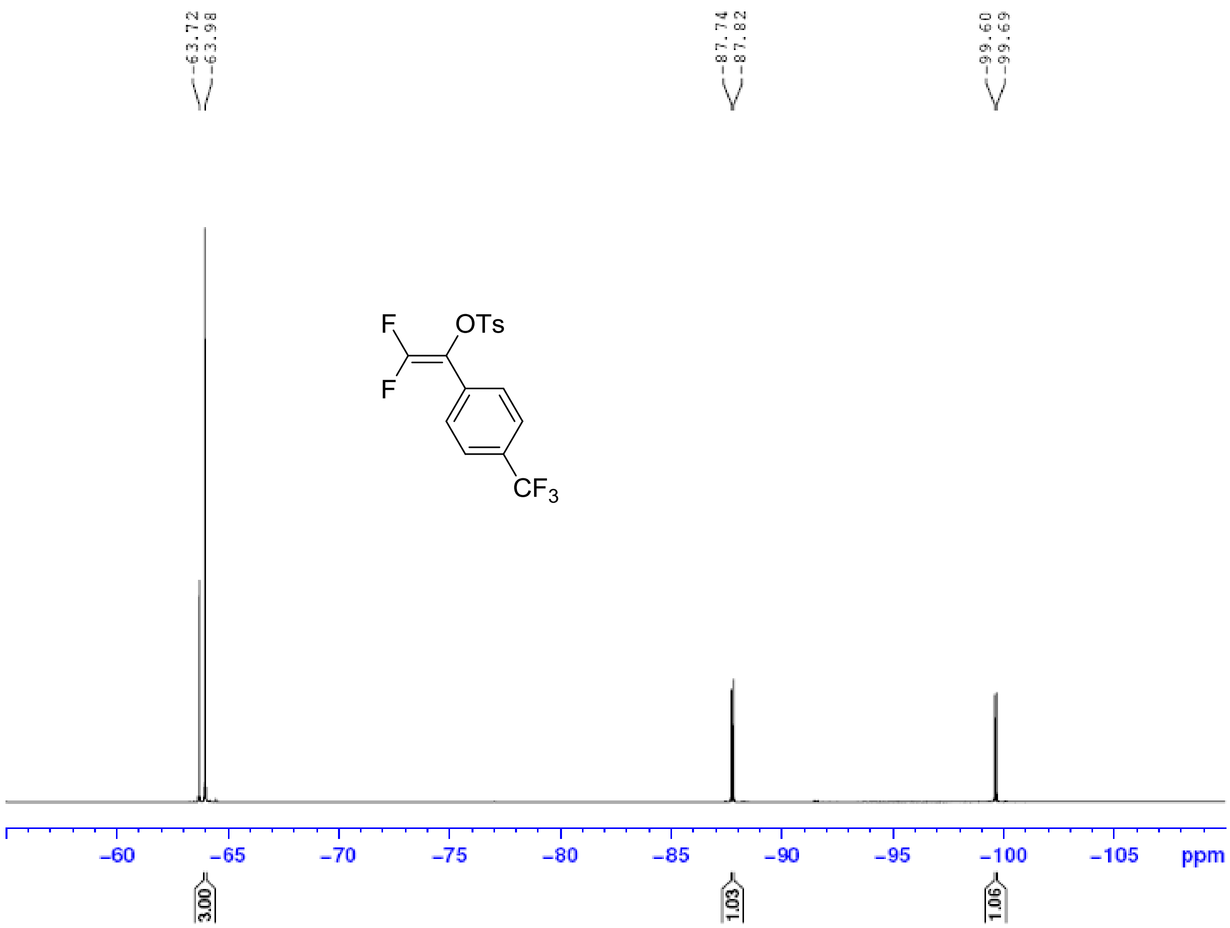

`

[¹H NMR spectrum of **3n**]

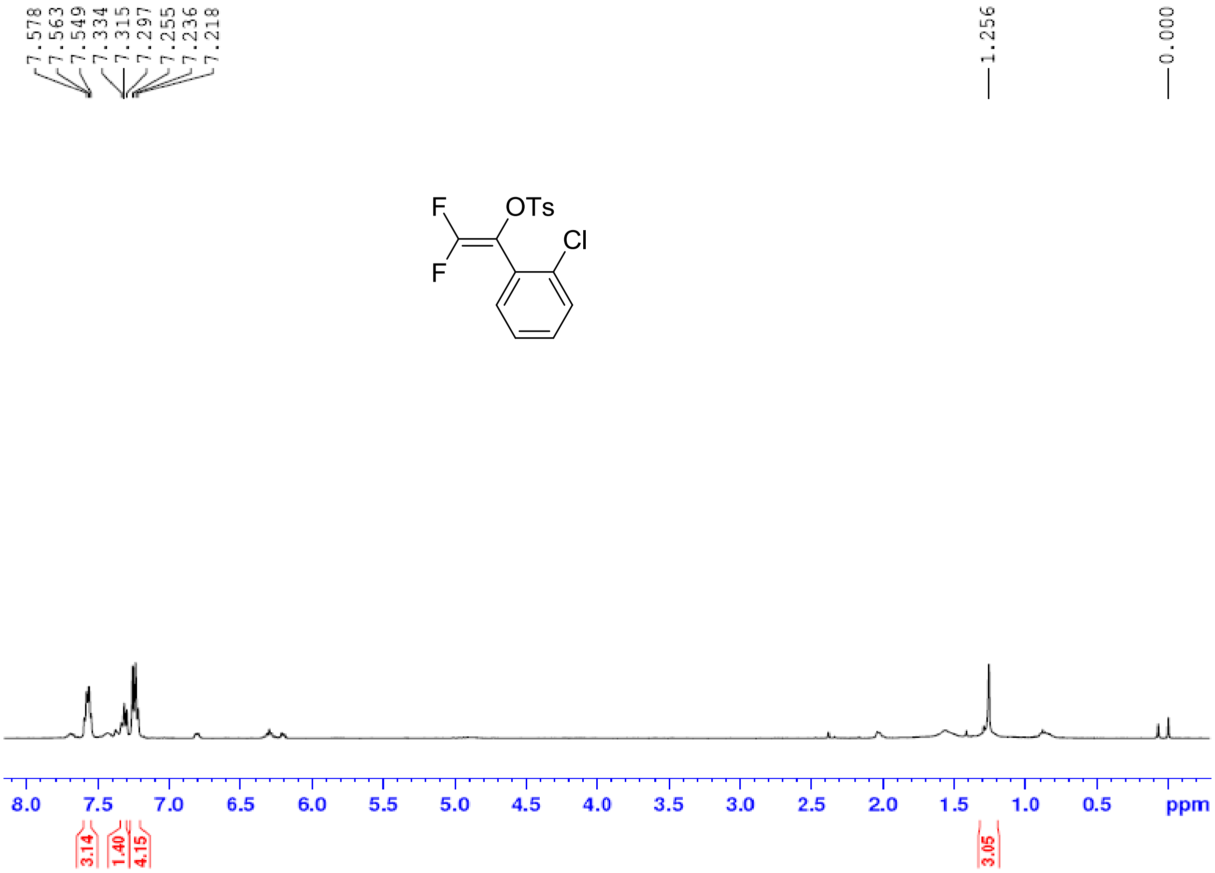

`

[^{13}C NMR spectrum of **3n**]

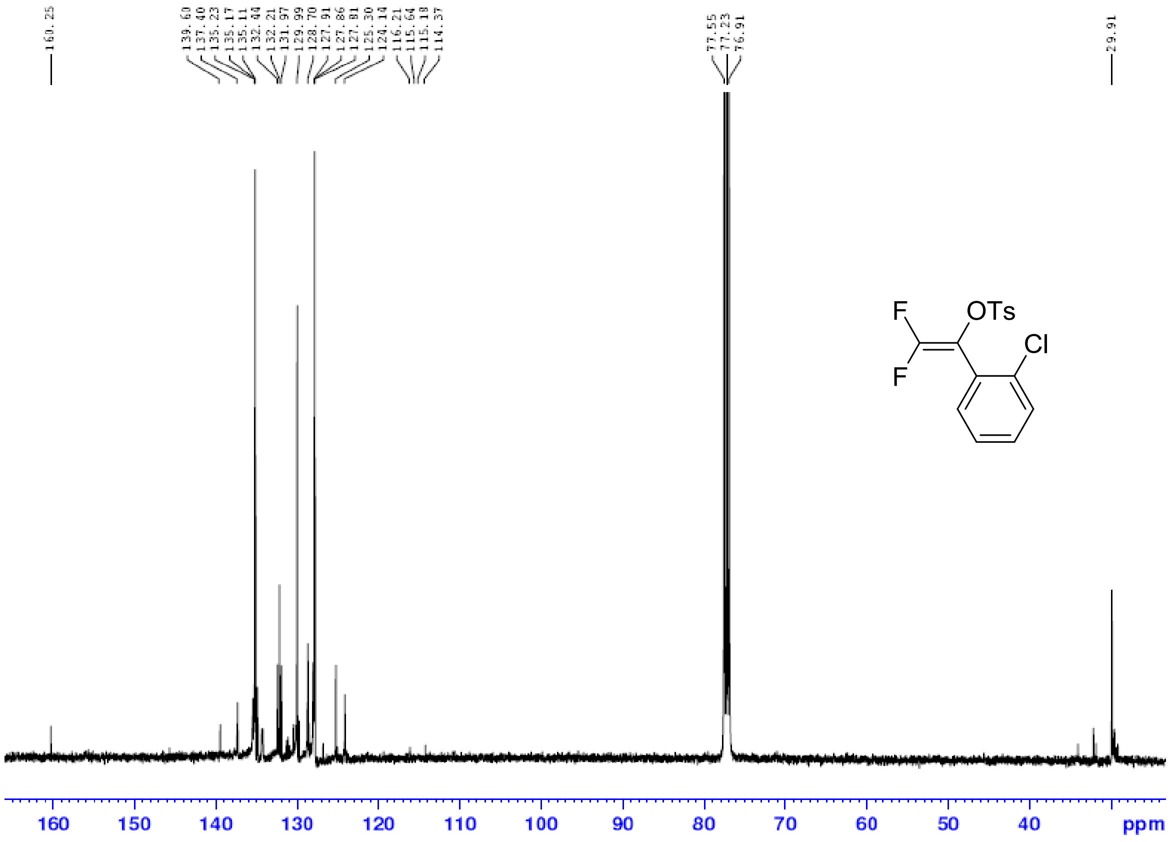

`

[^{19}F NMR spectrum of **3n**]

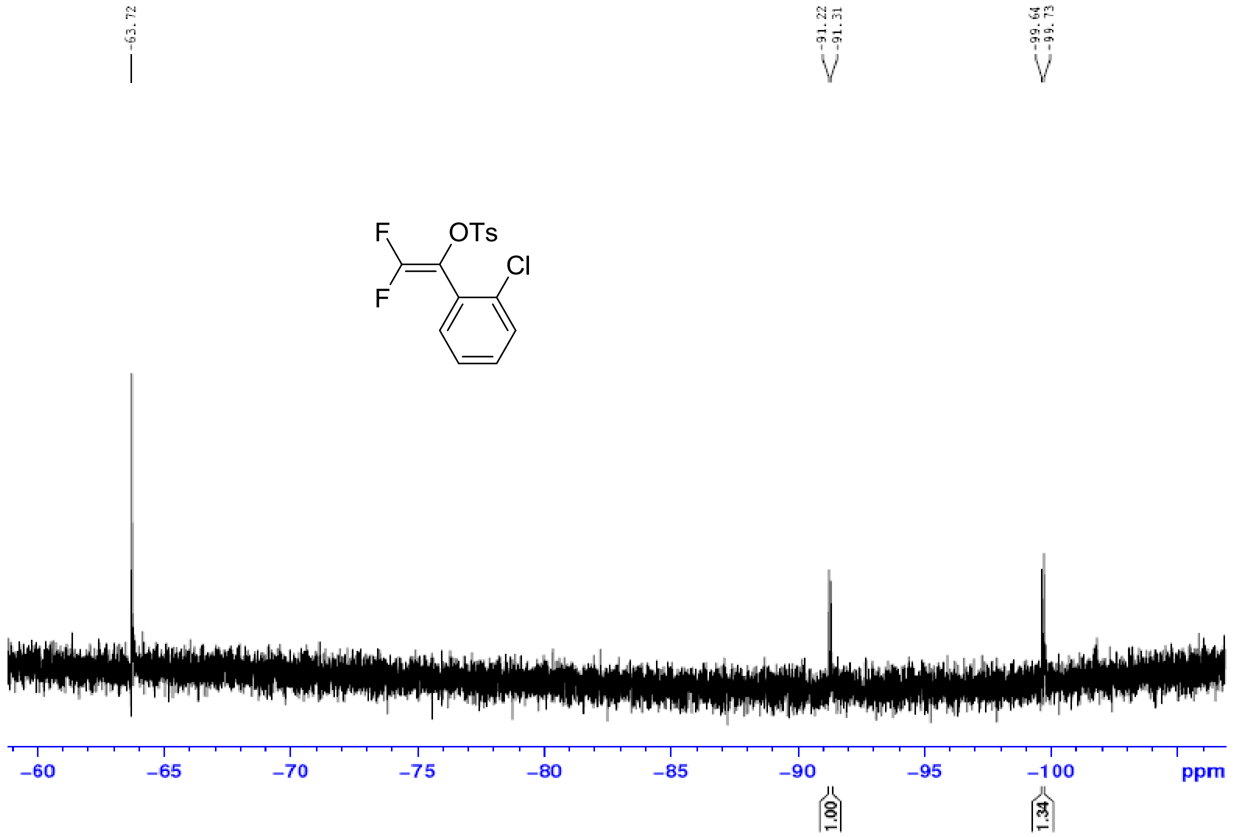

`

[^{1}H NMR spectrum of **4p**]

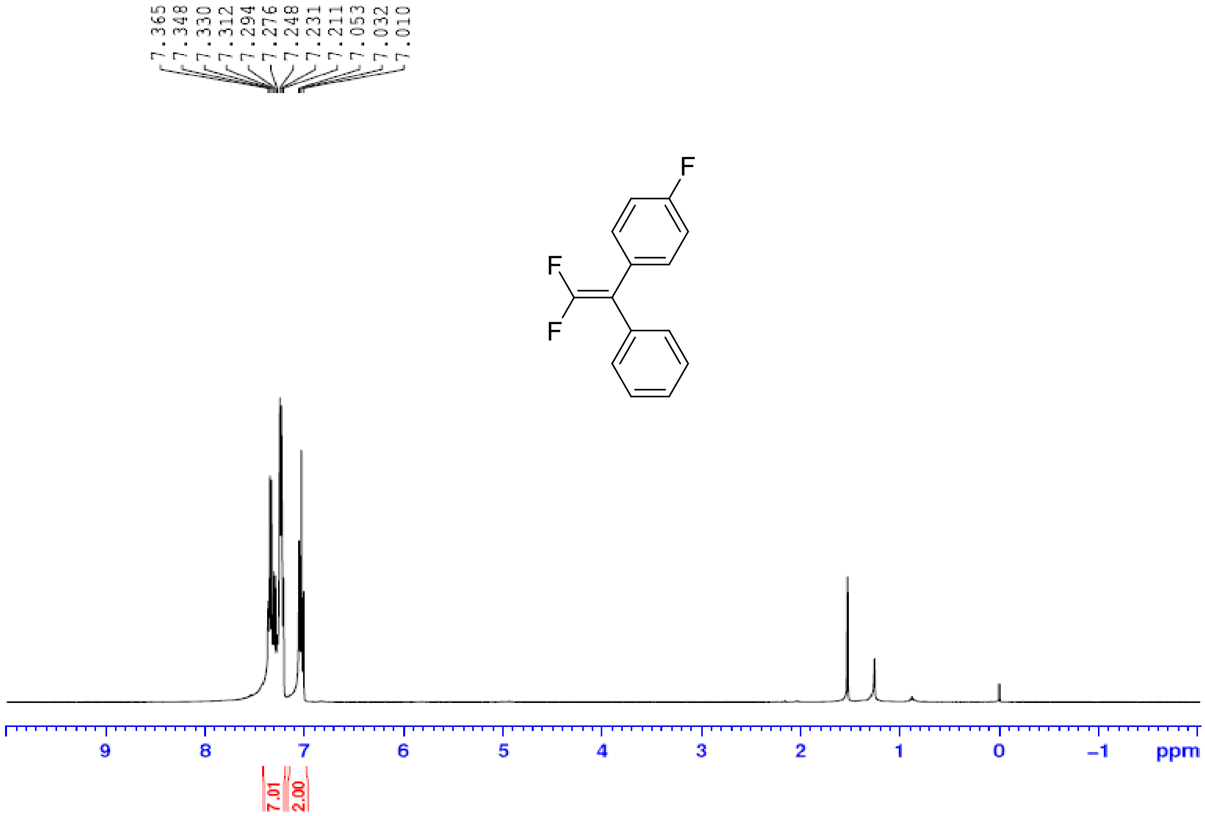

`

[^{13}C NMR spectrum of **4p**]

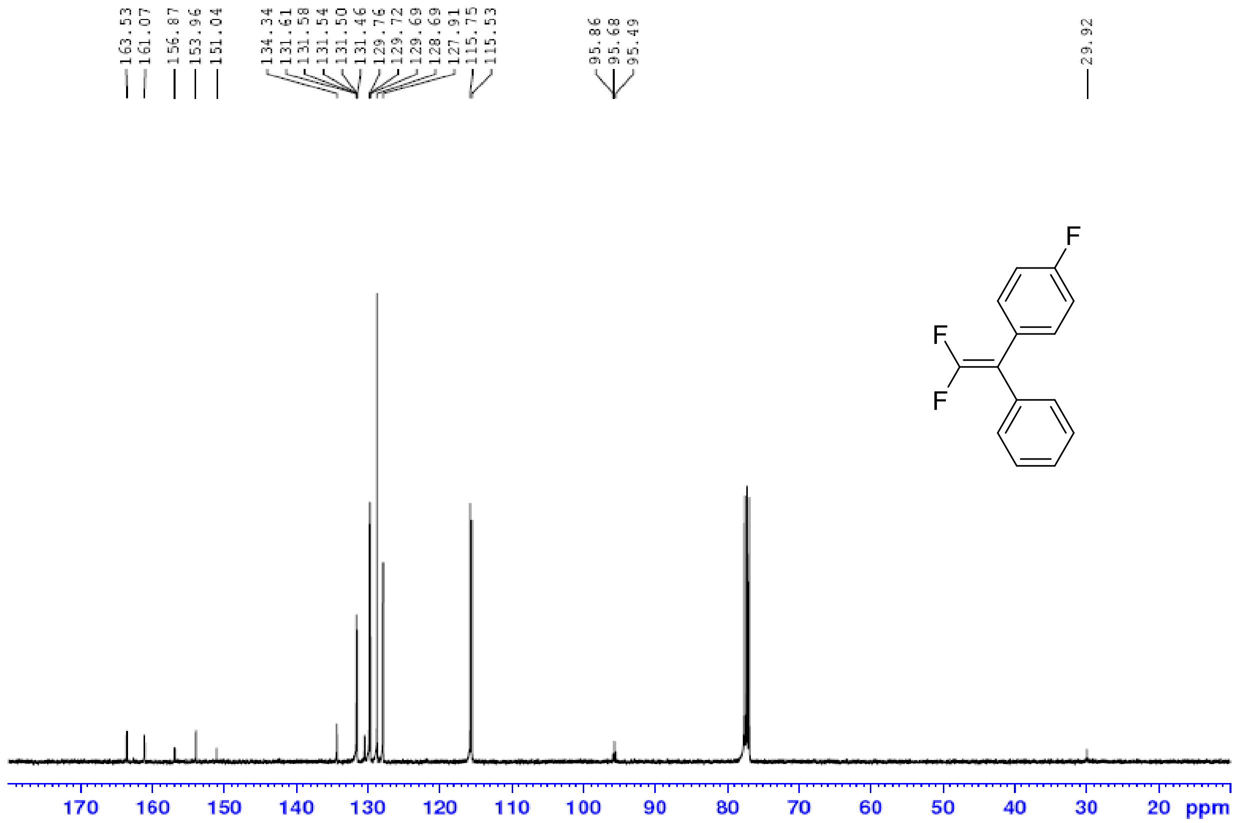

`

[^{19}F NMR spectrum of **4p**]

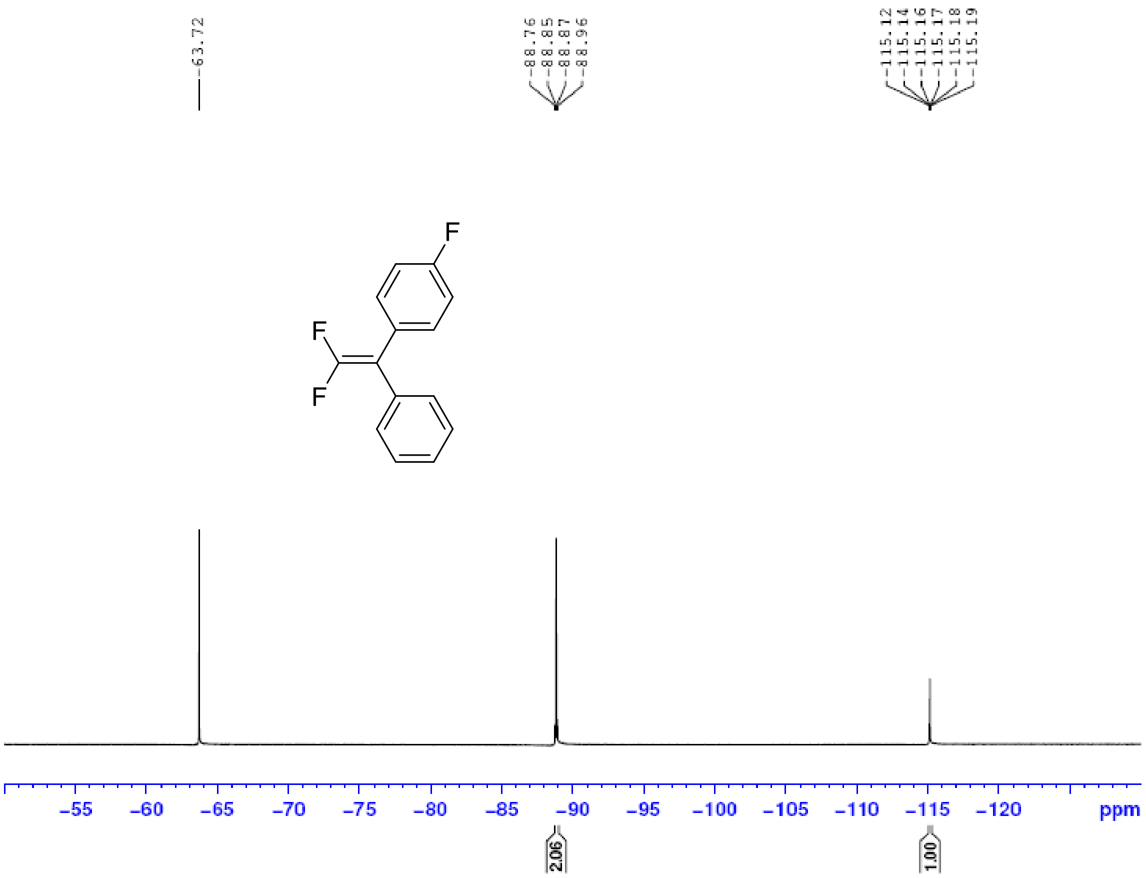

`

[^{1}H NMR spectrum of **4s**]

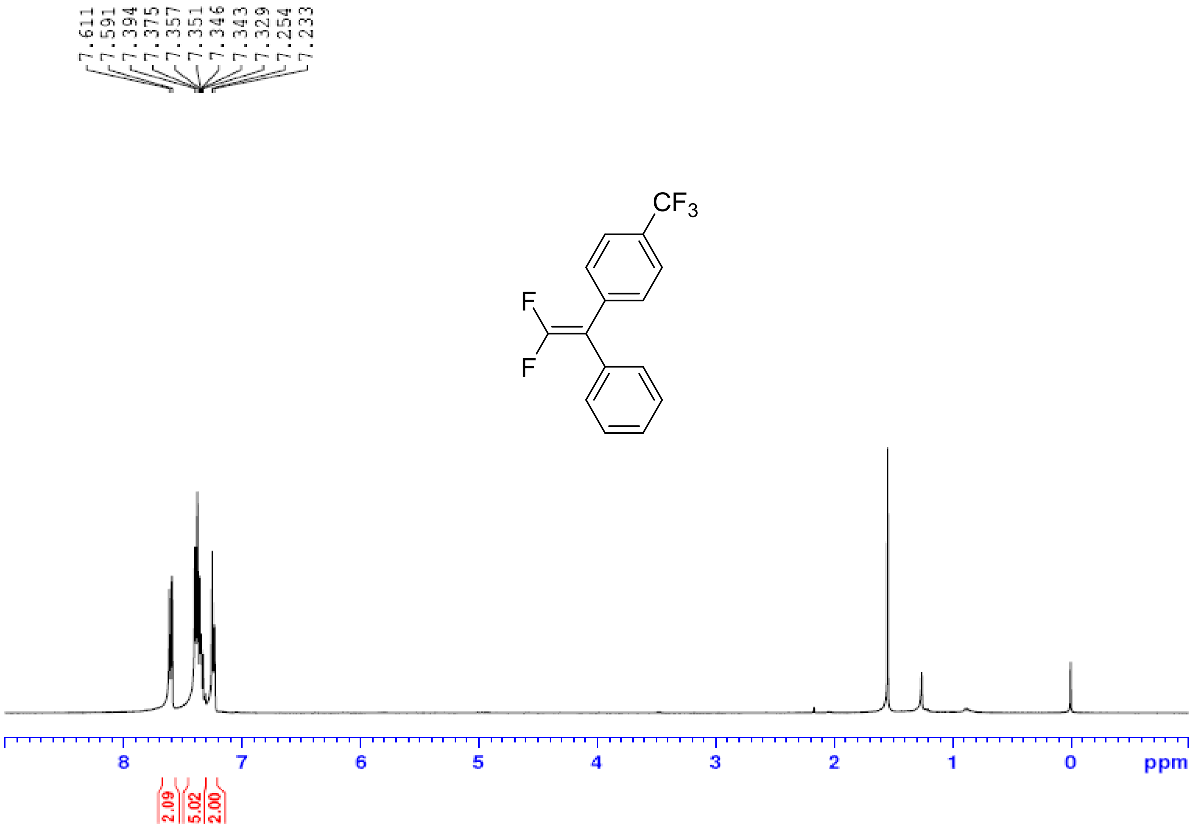

`

[^{13}C NMR spectrum of **4s**]

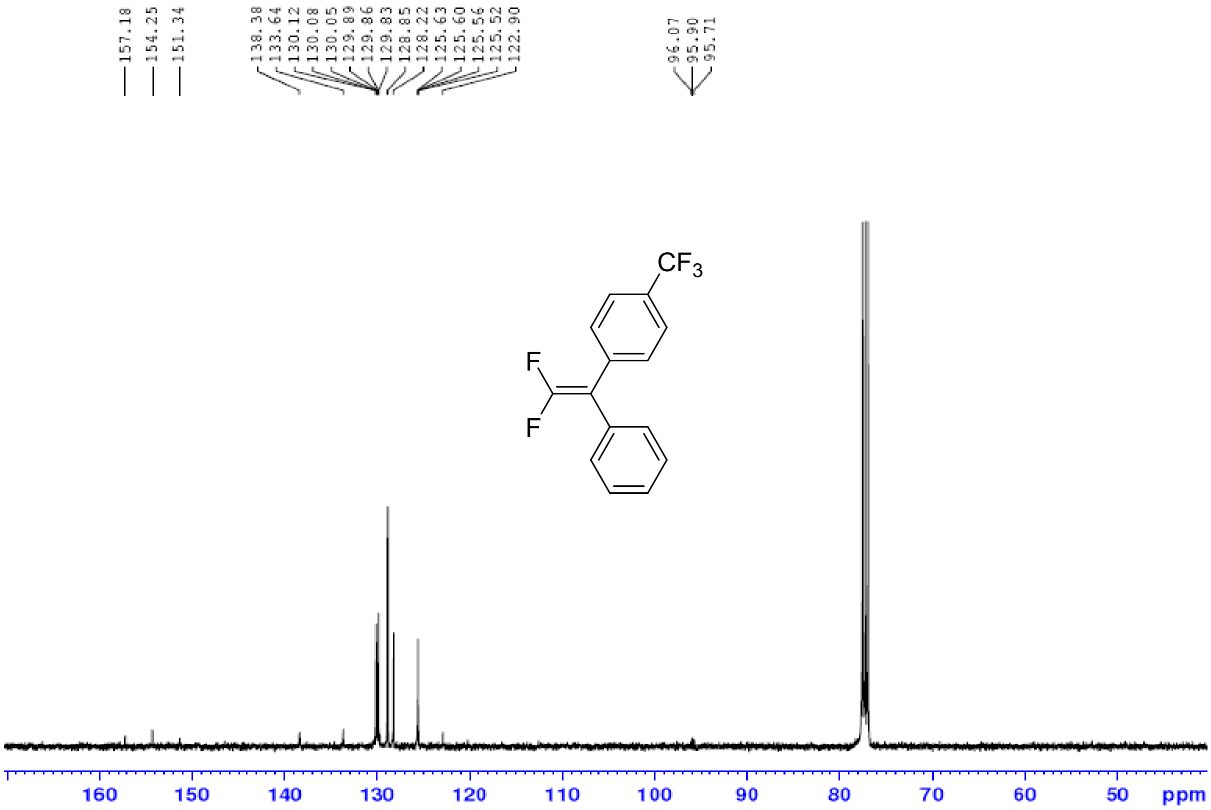

`

[^{19}F NMR spectrum of **4s**]

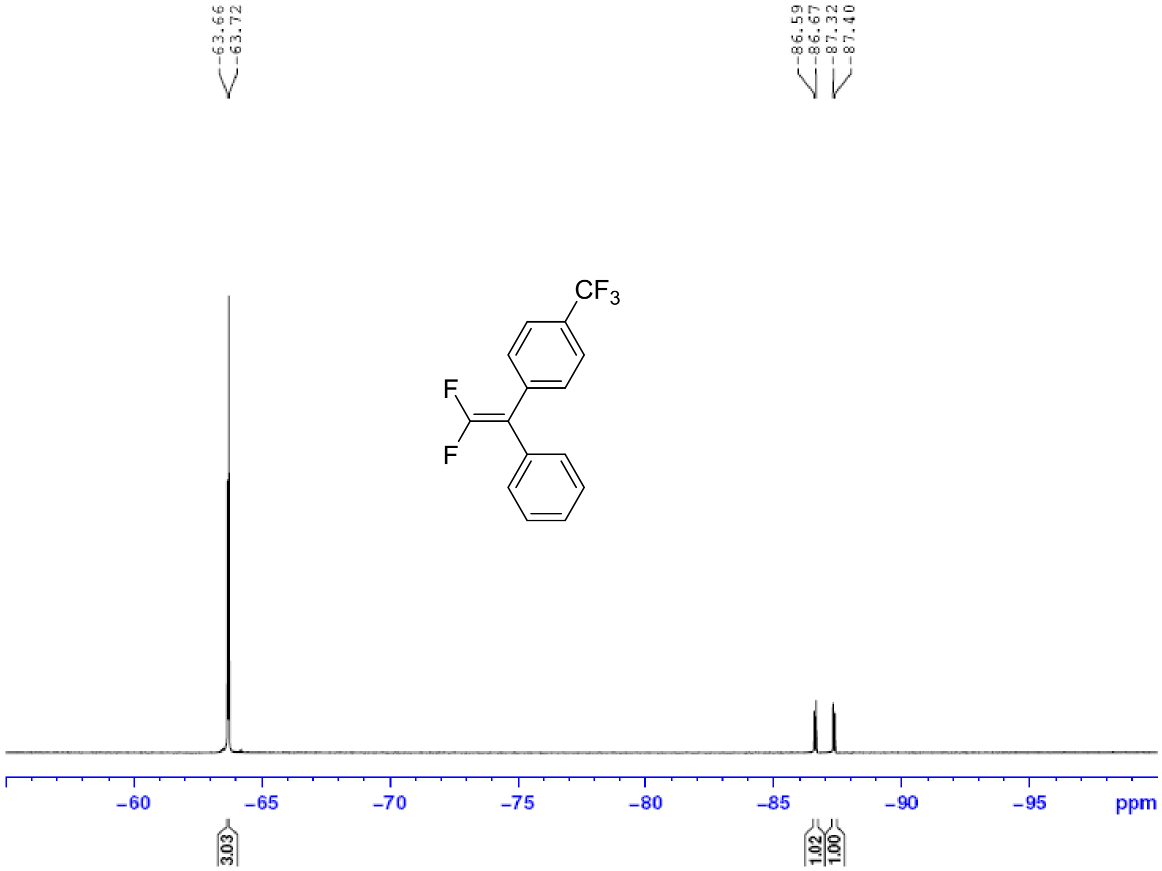

`

[^{1}H NMR spectrum of **4t**]

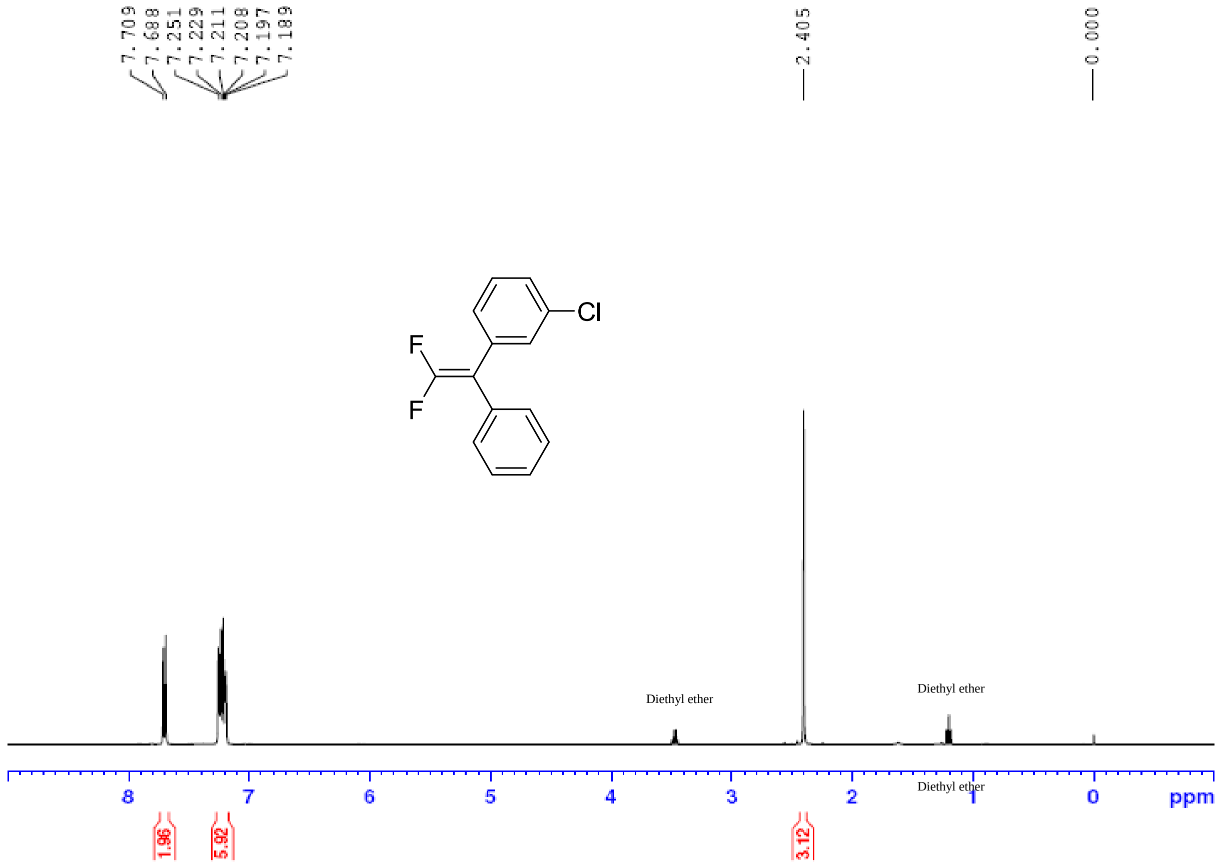

`

[^{13}C NMR spectrum of **4t**]

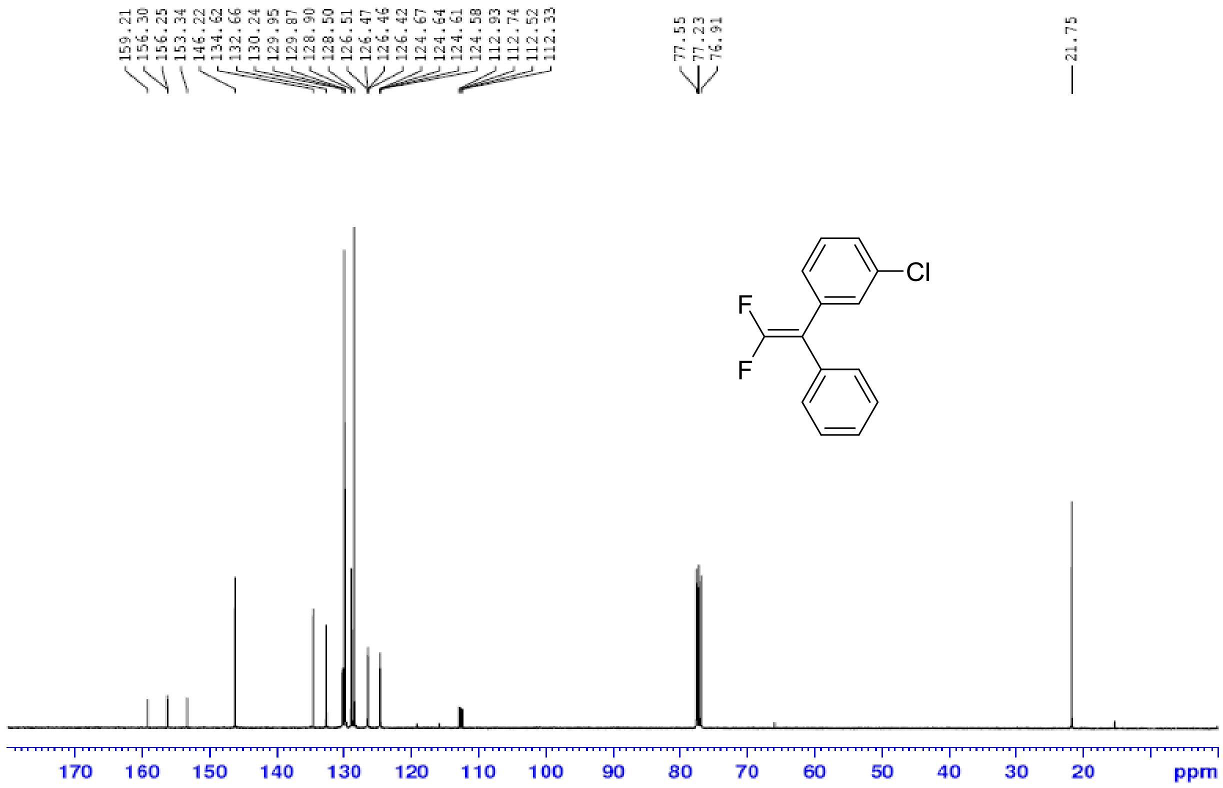

`

[^{19}F NMR spectrum of **4t**]

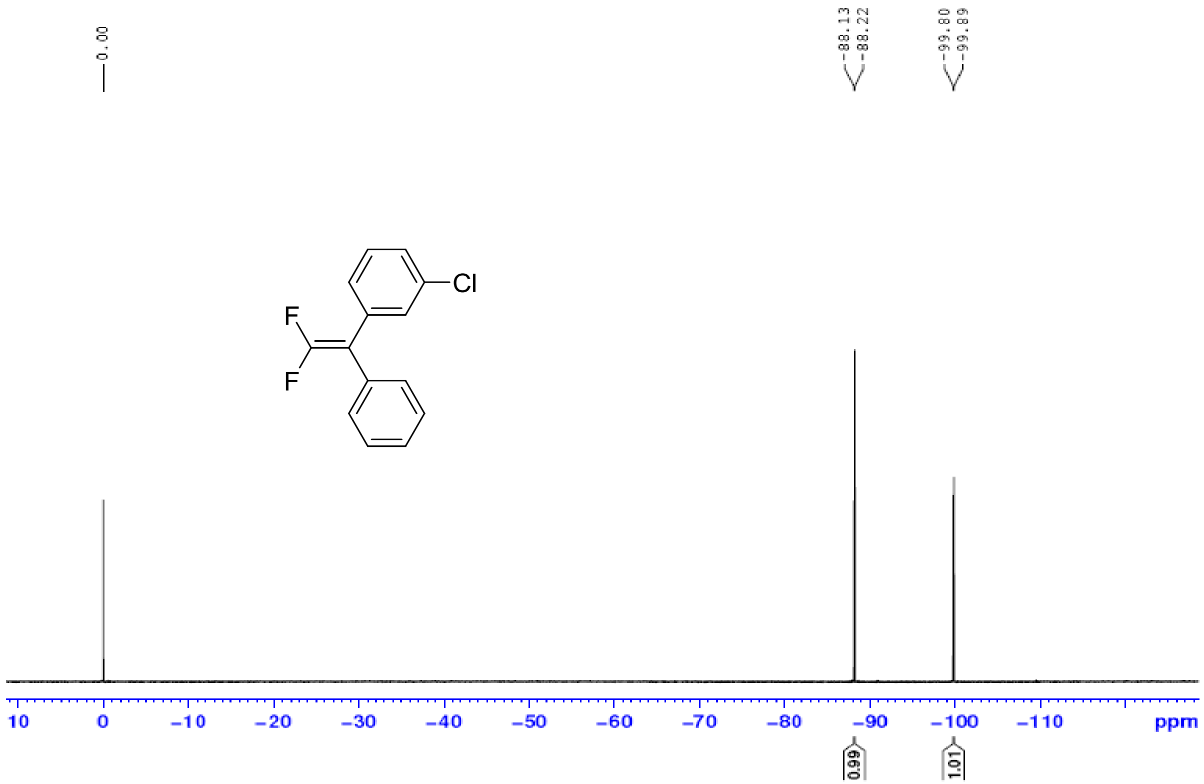

`

[^{1}H NMR spectrum of **4x**]

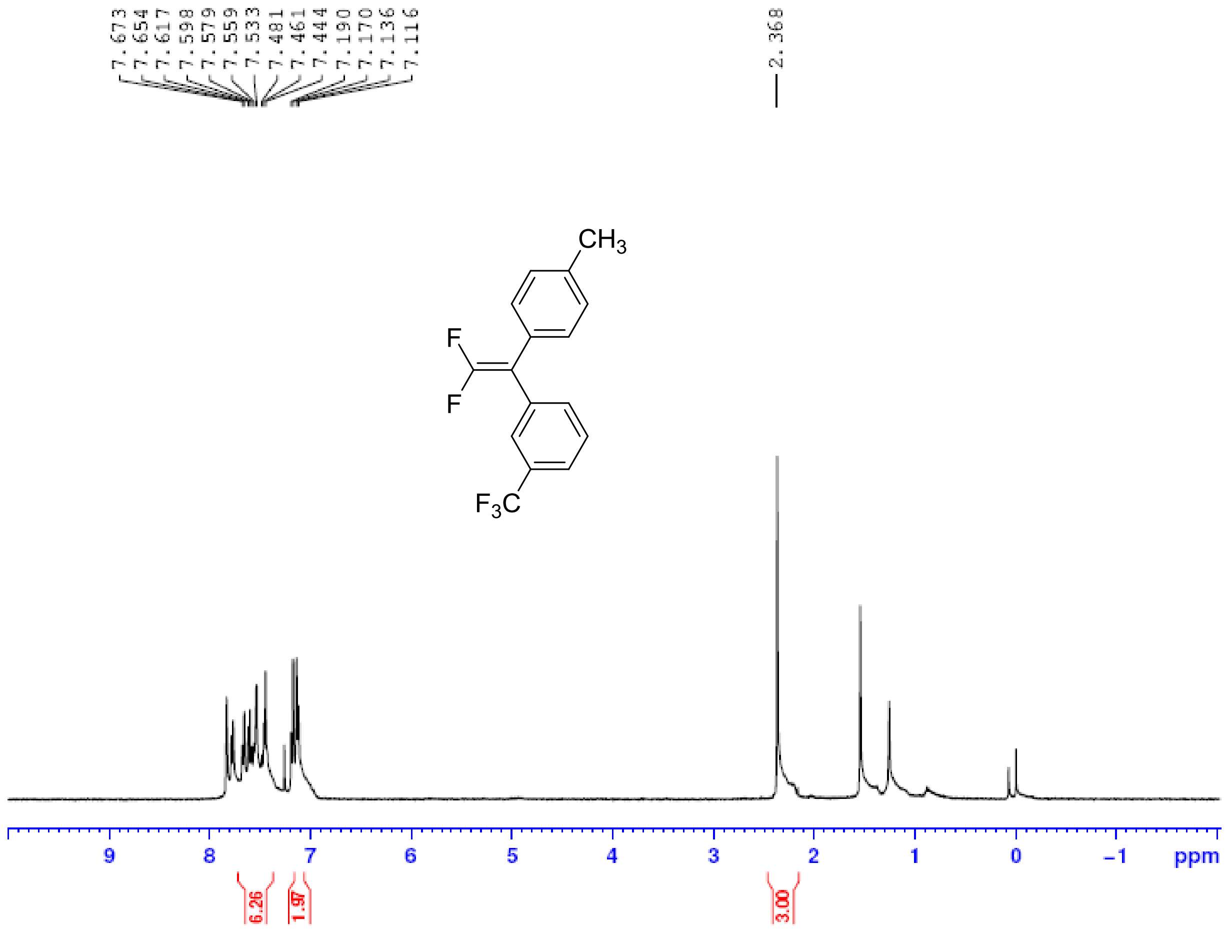

`

[^{13}C NMR spectrum of **4x**]

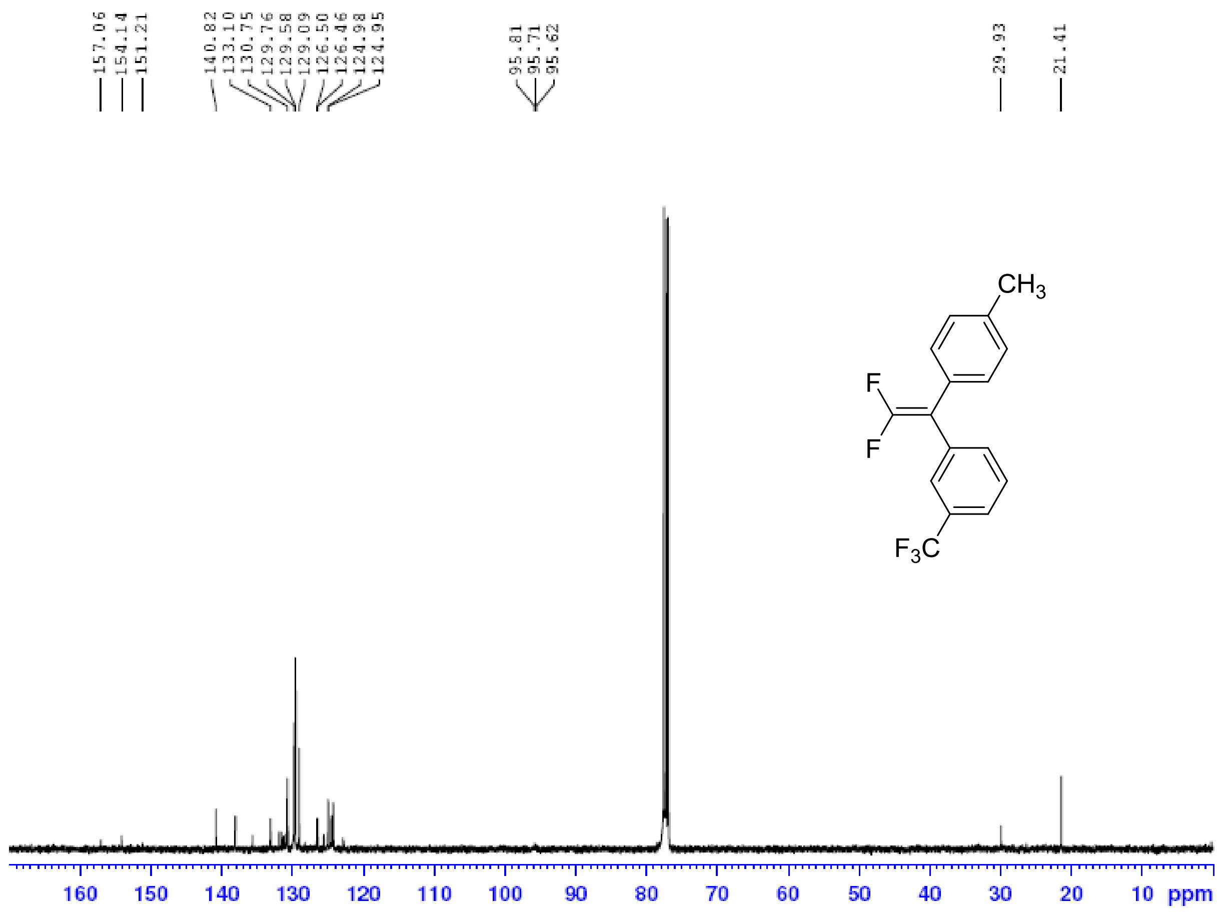

`

[^{19}F NMR spectrum of **4x**]

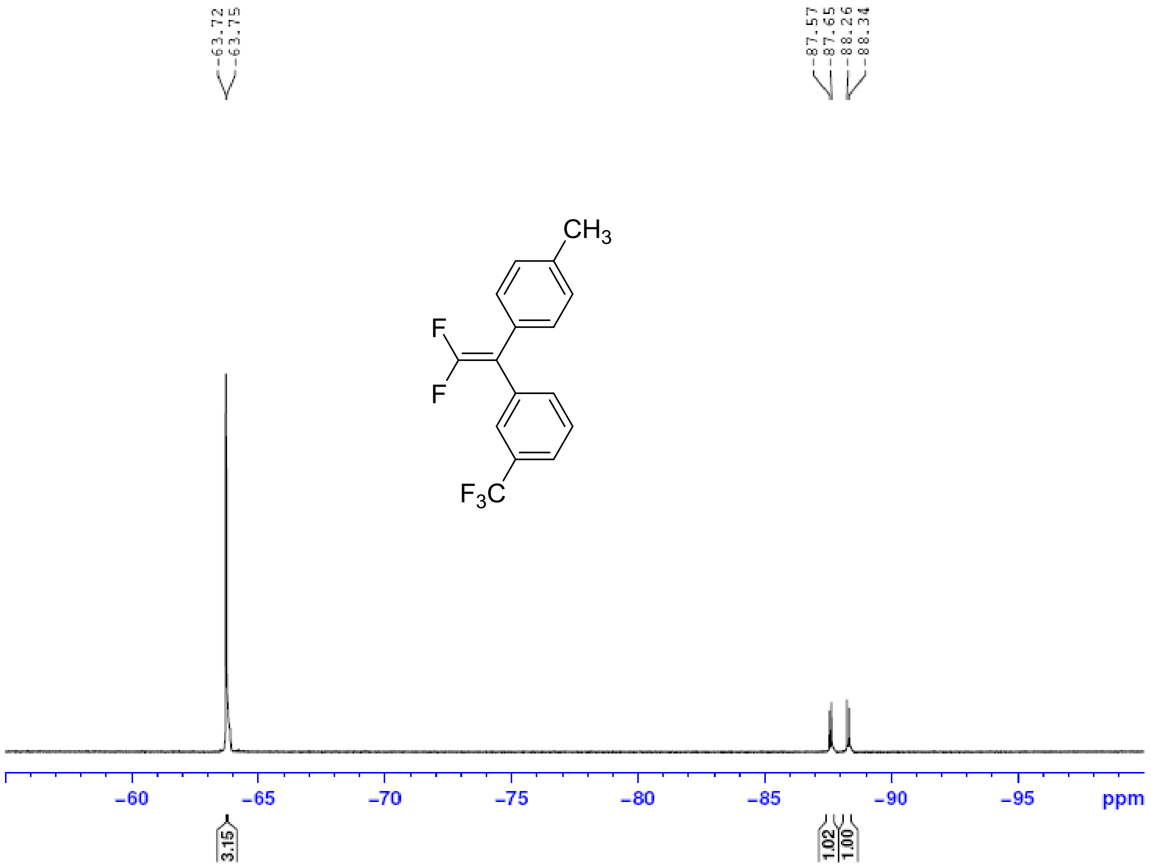

`

[^{1}H NMR spectrum of **5a**]

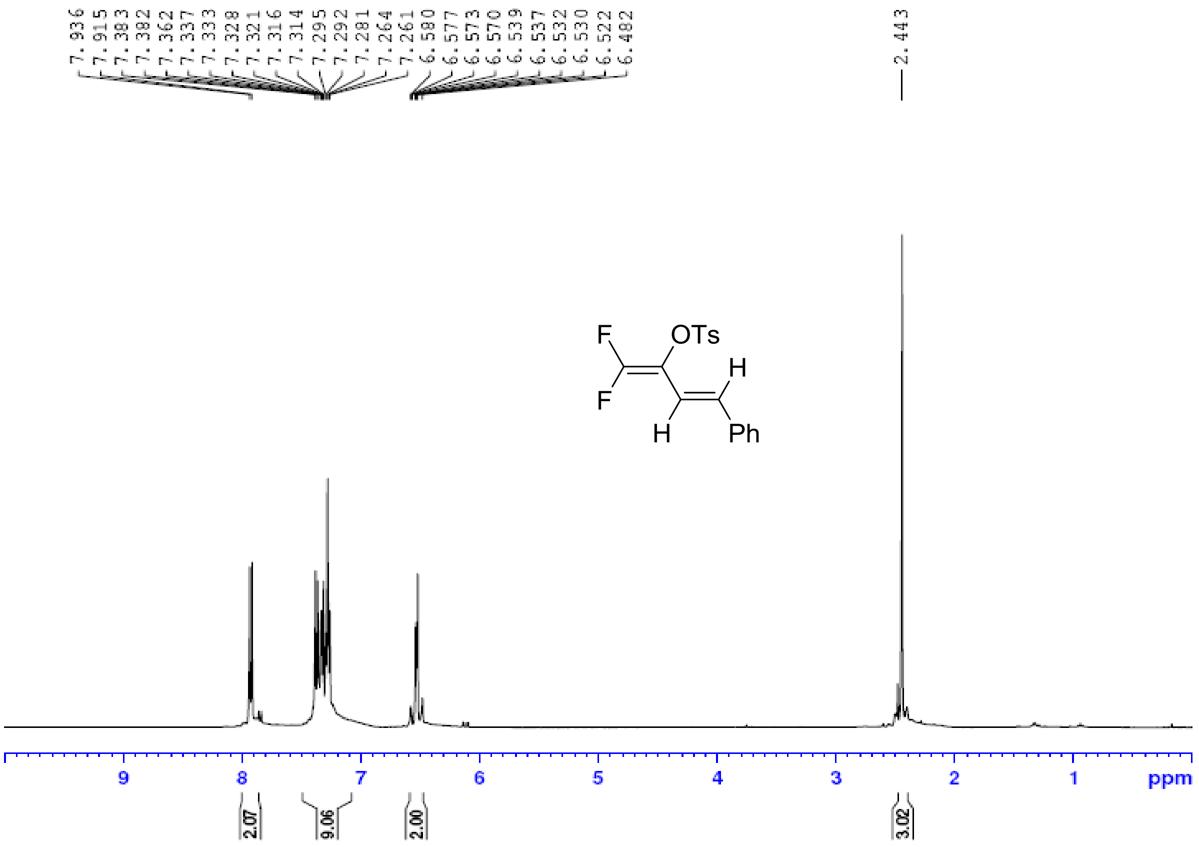

`

[^{13}C NMR spectrum of **5a**]

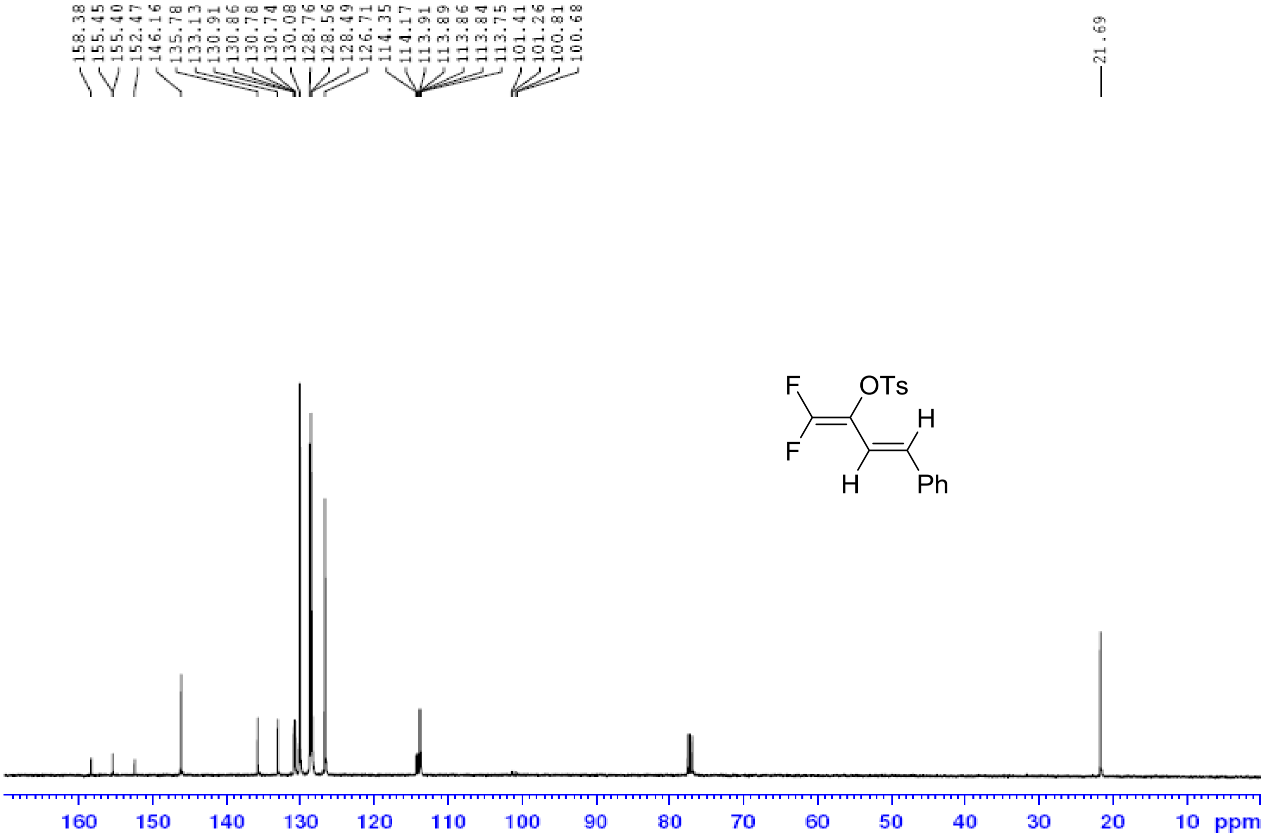

`

[^{19}F NMR spectrum of **5a**]

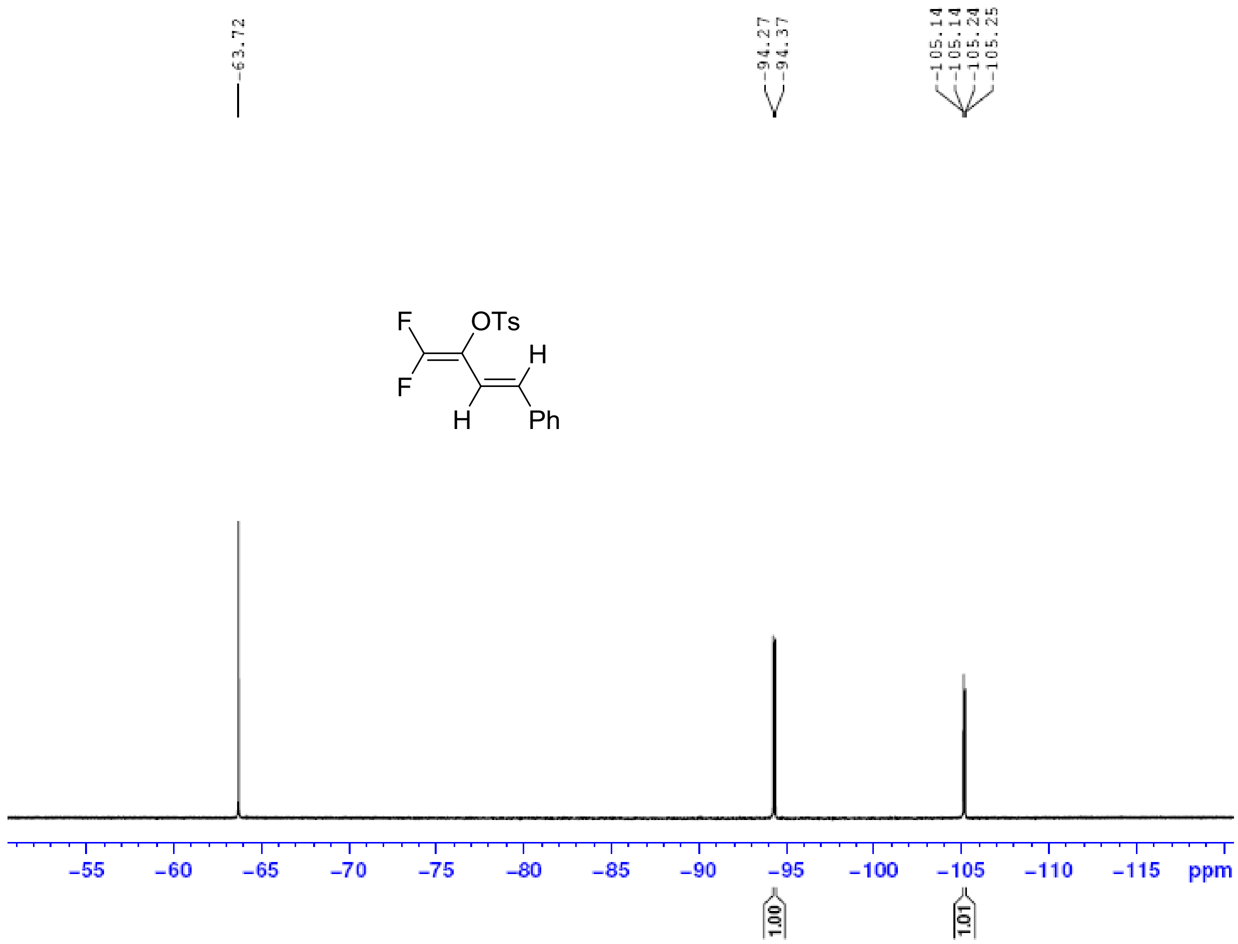

`

[^{1}H NMR spectrum of **5b**]

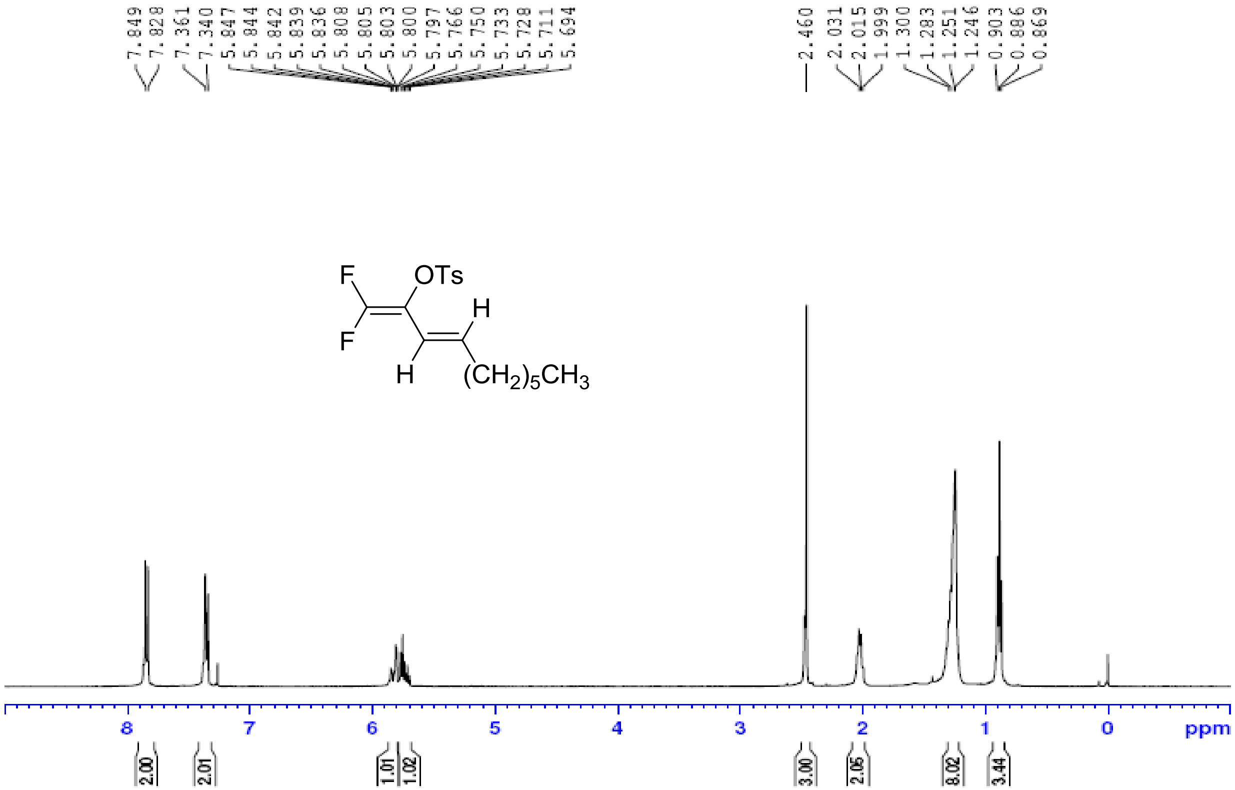

`

[13C NMR spectrum of **5b**]

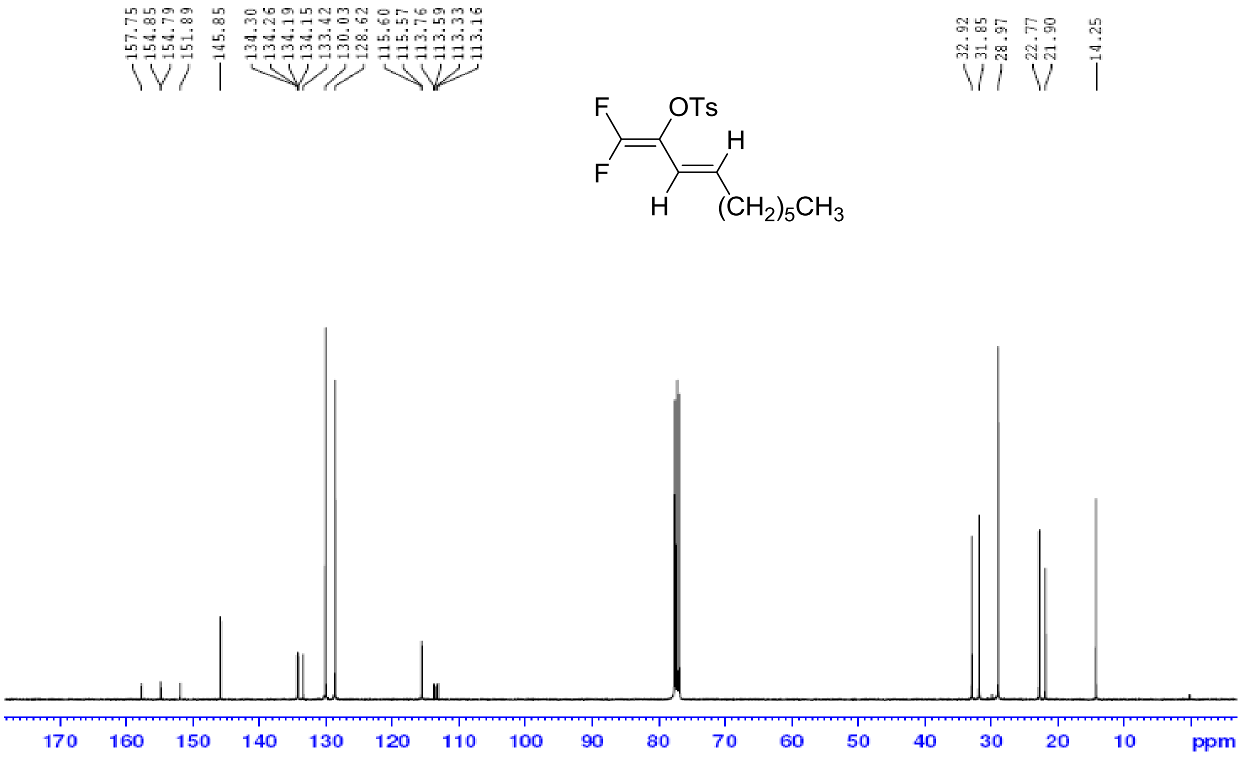

`

[^{19}F NMR spectrum of **5b**]

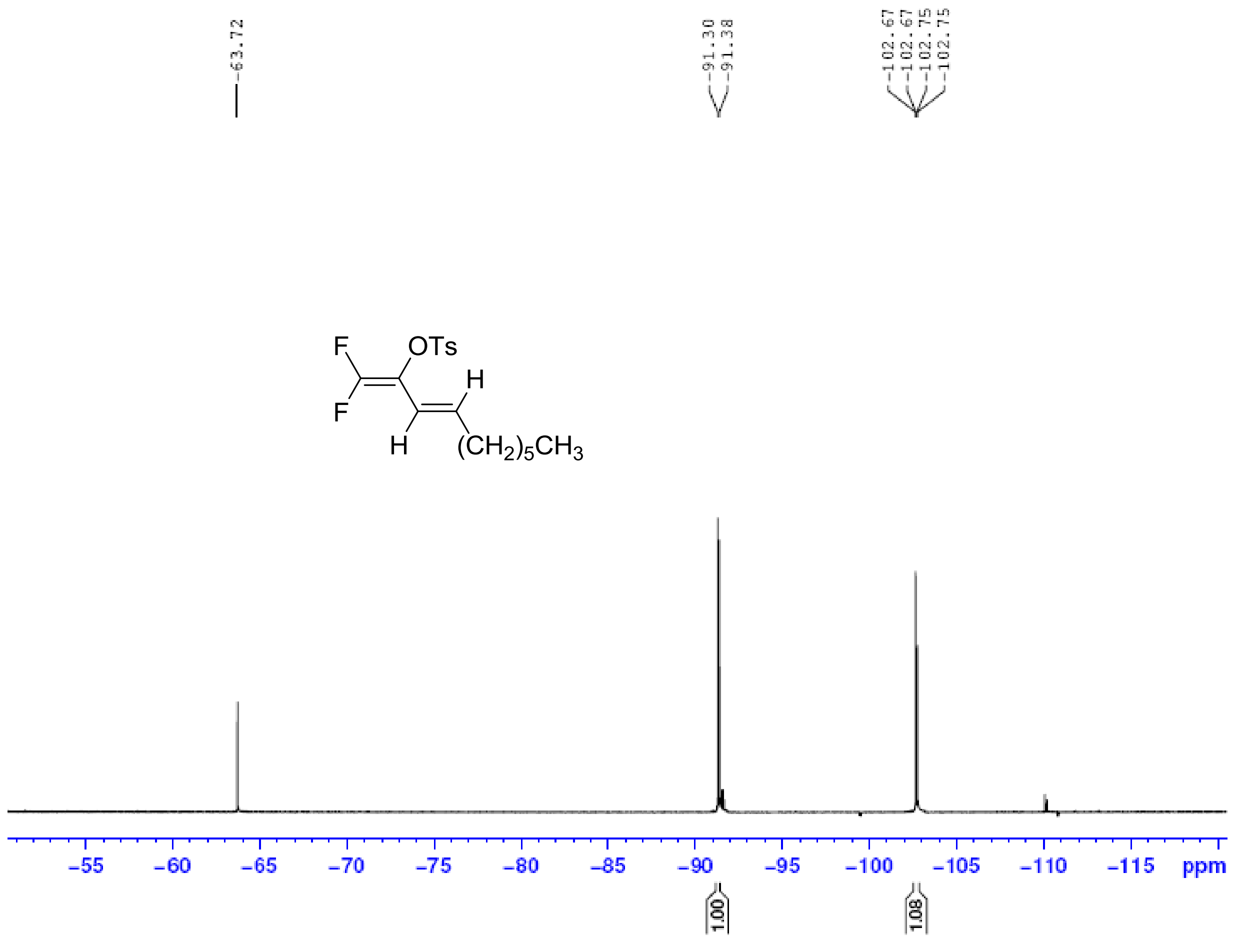

References:

1. Han, S. Y.; Jeong, I. H. *Org. Lett.* **2010,** *12,* 5518–5521. doi:10.1021/ol1024037

2. Han, S. Y.; Lee, H. Y.; Jeon, J. H.; Jeong, I. H. *Tetrahedron Lett.* **2012,** *53,* 1833–1836. doi:10.1016/j.tetlet.2012.01.127
